# How does exposure to pesticides vary in space and time for residents living near to treated orchards?

**DOI:** 10.1007/s11356-017-0064-5

**Published:** 2017-09-25

**Authors:** Hie Ling Wong, David G. Garthwaite, Carmel T. Ramwell, Colin D. Brown

**Affiliations:** 1 Environment Department, University of York, York, YO10 5NG UK; 2 Faculty of Earth Science, University Malaysia Kelantan, Locked Bag 100, 17600 Jeli, Kelantan Malaysia; 3 Fera Science Ltd (Fera), Sand Hutton, York, YO41 1LZ UK

**Keywords:** Pesticide, Usage, Orchard, Resident, Exposure, Risk, Regulation

## Abstract

This study investigated changes over 25 years (1987–2012) in pesticide usage in orchards in England and Wales and associated changes to exposure and risk for resident pregnant women living 100 and 1000 m downwind of treated areas. A model was developed to estimate aggregated daily exposure to pesticides via inhaled vapour and indirect dermal contact with contaminated ground, whilst risk was expressed as a hazard quotient (HQ) based on estimated exposure and the no observed (adverse) effect level for reproductive and developmental effects. Results show the largest changes occurred between 1987 and 1996 with total pesticide usage reduced by ca. 25%, exposure per unit of pesticide applied slightly increased, and a reduction in risk per unit exposure by factors of 1.3 to 3. Thereafter, there were no consistent changes in use between 1996 and 2012, with an increase in number of applications to each crop balanced by a decrease in average application rate. Exposure per unit of pesticide applied decreased consistently over this period such that values in 2012 for this metric were 48–65% of those in 1987, and there were further smaller decreases in risk per unit exposure. All aggregated hazard quotients were two to three orders of magnitude smaller than one, despite the inherent simplifications of assuming co-occurrence of exposure to all pesticides and additivity of effects. Hazard quotients at 1000 m were 5 to 16 times smaller than those at 100 m. There were clear signals of the impact of regulatory intervention in improving the fate and hazard profiles of pesticides used in orchards in England and Wales over the period investigated.

**Electronic supplementary material:**

The online version of this article (10.1007/s11356-017-0064-5) contains supplementary material, which is available to authorized users.

## Introduction

Pesticides are bioactive substances that have been widely used to improve agricultural production, reduce yield losses and maintain high product quality in order to meet the increasing demand for food from the world’s growing population, particularly in intensive agricultural systems. Pesticides are chemical or biological agents designed to kill potential disease-causing organisms and control insects, other pests and weeds in both open and protected environments. Due to their intrinsic toxicity, it is necessary to quantify potential for transportation away from the point of application, exposure to humans and non-target ecosystems, and risk to human and ecological health. Pesticides are amongst the most highly regulated chemical classes due to the combination of bioactivity and use in open environments.

Spray drift and volatilisation followed by transport in the vapour phase are potential routes for dispersal of pesticides via the air. Spray drift is the downwind movement of spray droplets beyond the treated area at the time of application or soon after (Felsot et al. 2010). It is influenced by the nozzle and operating pressure of the equipment, height of the spray boom, and weather conditions at the time of application (Hofman and Solseng 2001). After an application is complete, volatilisation followed by transport in the vapour phase can be an important pathway for pesticide emission from treated soil and plant surfaces, at the extreme accounting for as much as 90% of the applied dose over a period of a few days to several weeks (Bedos et al. 2002; Lichiheb et al. 2014). Sarigiannis et al. (2013) proposed that volatilisation from plant surfaces can be up to three times greater than that from soil, and volatilisation can be more important for total emissions of active substances compared to spray drift in the long term.

After entering into the atmosphere, spray drift can be transported by the wind before deposition of spray droplets locally whilst pesticide in the vapour phase following volatilisation can be transported over longer distances (Briand et al. 2002). Whilst much work has been done to measure downwind deposition of spray droplets, there is a lack of consistent methodology for quantifying airborne pesticide concentrations at a range of scales (Zivan et al. 2016; Lichiheb et al. 2016). Mathematical models are useful in complementing expensive and time-consuming field trials by including the complex processes that mediate the transfer of pesticides between different environmental compartments (Salcedo et al. 2017). A number of previous studies calculated vapour exposure using volatilisation models coupled with different dispersion modelling approaches including 3D Gaussian and a 2D version of OPS (Operational Atmospheric Transport Model for Priority Substances) (van den Berg et al. 2016). The BROWSE model (Bystanders, Residents, Operators and WorkerS Exposure models for plant protection products) is a recent development that combines a mechanistic volatilisation model and an advanced 3D dispersion model of OPS (van den Berg et al. 2016). Development of models for aerial transport and exposure to pesticides is still restricted by data availability. For example, the best data available whilst developing the airborne spray component of the BROWSE’s orchard model did not give sufficient confidence in quantifying spray drift under different meteorological conditions and at different distances of exposure, implying that further experimental data are needed (Butler Ellis et al. 2017).

There is evidence to suggest that residents living close to agricultural fields have greater exposure to pesticides compared to the general population, but very few studies have examined the dose-response relationships between exposure and health outcomes of interest (Shirangi et al. 2010). Sensitive sub-populations amongst residents could be at higher risk of health impacts than the general population and include foetuses, children, pregnant and nursing mothers, and the elderly (Costa et al. 2014). A systematic review and meta-analysis on residential exposure to pesticides and childhood leukaemia for 13 case-control studies published between 1987 and 2009 indicated stronger risk for exposure during pregnancy (meta-rate ratio (mRR): 2.19, 95% confidence intervals (CI): 1.92–2.50) compared to after pregnancy (mRR: 1.65, 95% CI: 1.33–2.05) (Van Maele-Fabry et al. 2011). Nevertheless, the study highlighted recall bias as a major limitation of case-control studies where questionnaire data are used to assess past exposure. Shirangi et al. (2010) suggested that residential proximity to pesticide applications during pregnancy could be associated with adverse reproductive outcomes in offspring. However, epidemiological evidence from 25 studies published between 1950 and 2007 was generally weak, primarily due to limitations in the assessment of exposure. The study suggested that future research should refine the methods on exposure modelling by incorporating environmental monitoring studies on pesticide drift. Weselak et al. (2007) reviewed epidemiological evidence on periconceptual pesticide exposures and developmental outcomes based on studies published between 1966 and 2005 and reported generally poor exposure estimations and limited evidence for causality in all the associations examined due to self-reported, indirect, or proxy exposure measures.

Regulatory assessments prior to authorisation of plant protection products require quantitative estimates of exposure to pesticides via the air for comparison with toxicological reference levels, below which no adverse health effects are expected (Galea et al. 2015). In Europe, the estimation of exposure to pesticides for operators, workers, residents and bystanders is underpinned by the guidance of EFSA (2014). However, sparcity of data on concentrations of volatilised pesticides in air has been noted as a limitation on exposure assessment (Butler Ellis et al. 2010), as has a general lack of research into methods for estimating exposure and risk to the general public (Coscolla et al. 2017).

The Pesticide Authorisation Directive 91/414/EEC, ratified in 1993, legislated for a comprehensive review of plant protection products already on the market; of the ca. 1000 active substances on the market in 1993 in at least one Member State, only around 250 (26%) passed the EU harmonised safety assessment, with the remainder either unsupported by industry (67%) or rejected following review (7%) (Balderacchi and Trevisan 2010; European Commission 2009). These pesticides were mainly deregistered due to either their toxicity profile or restricted efficacy due to the development of resistance in the control target (Karabelas et al. 2009).

Post-authorisation monitoring schemes provide an important check that regulatory procedures are robust in the protection afforded to human health. In the UK, the Pesticide Incidents Appraisal Panel (PIAP) of the Health and Safety Executive (HSE) reviews incidents of alleged ill health that are attributed to pesticide exposure both at work and for members of the public (HSE 2015). The Pesticide Incident Report 2012/13 (HSE 2015) investigated 45 pesticide incidents (64% lower than the average for the previous 10 years), with 15 complaints involving allegations of ill health of which 20–25% were classified as ‘confirmed’ or ‘likely’. An earlier scheme based on general practitioners estimated the prevalence and incidence of pesticide-related illness between 2004 and 2008. That study identified significant limitations in defining a pesticide-related cause of ill health because there is generally limited information on actual chemicals used and no routine confirmation of exposure through biological tests (Rushton and Mann 2008). These are important caveats on the overall conclusion from post-authorisation monitoring that there is no evidence for widespread impacts of agricultural pesticides on human health in the UK.

Whilst much work considers the risks to human health from use of pesticides, there is a gap between risk assessment as part of regulatory procedures, post-authorisation monitoring, and longer-term epidemiological investigations. Regulatory assessments are the only place where exposure is routinely quantified, but this is done one chemical at a time and there is no oversight of total exposure to pesticides or of how this may be changing in time. Post-authorisation monitoring and epidemiological studies take a more holistic perspective on potential for health impacts, but have generally failed to include quantitative estimates of exposure. Thus an independent study of how exposure to pesticides varies in space and time provides an important check for the regulatory process.

This study investigates how pesticide usage and associated exposure and risk vary in space and time to provide a holistic evaluation of the impact of regulation. We selected off-target exposure to residents living close to treated areas as our test system, focusing on orchards which have relatively high usage of pesticides and treatments that are often directed into crop canopies, and pregnant women who are a vulnerable group because they may spend long periods at home and because some pesticides have potential for reproductive and/or developmental effects. We assessed variation in pesticide usage, exposure and risk (i) between orchard crops, (ii) between regions of England and Wales, (iii) across different seasons, and (iv) between different years over a time series spanning 25 years (1987–2012).

## Method

### Identification of potential routes/pathways of exposure

Cornelis et al. (2009) developed a GIS-based indicator for environmental exposure to pesticides, proposing the selection of cut-off values for the radii of zones around the site of application based on the decrease in airborne concentrations of pesticides. Following this procedure, two categories of proximity were identified in the current study, namely 0–200 m (central point at 100 m) and 0–2000 m (central point at 1000 m) such that airborne pesticide concentrations decreased by approximately 5-fold from 100 to 1000 m.

Off-target movement of pesticides can result in contaminated food, water, air, dust, and soil and the potential for human exposure via inhalation, ingestion or dermal absorption through contact with contaminated surfaces (Sutton et al. 2011). Four pathways of exposure are considered in the standard EU risk assessment for residents which uses a model of residents living 8 m downwind from the middle of the last row in orchard crops (EFSA 2014); these pathways are (i) spray drift resulting in direct exposure via dermal penetration and inhalation; (ii) spray drift causing deposits on the ground and other surfaces leading to dermal exposure; (iii) vapour dispersal leading to inhalation of airborne pesticides following volatilisation from residues on soil and/or the treated crop; and (iv) entry into treated crops causing exposure through direct contact with surface residues. Spray drift decreases very rapidly with distance from the treated field (Rautmann et al. 1999) and preliminary modelling showed that direct dermal and inhalation exposure from spray drift were insignificant contributors to total exposure for residents living 100 or 1000 m from the treated area due to the combination of rapid fallout of spray droplets from the air with increasing distance from the site of application (Sarigiannis et al. 2013; van de Zande et al. 2014), and short duration of exposure. As direct exposure to airborne spray droplets occurs only at the time of application or soon after, residents are mainly exposed to pesticides via the indirect dermal route from spray drift deposits (e.g. working, standing or sitting in a garden near to the application) and inhaled pesticide vapour that may occur continuously throughout the day (Felsot et al. 2010; Martin et al. 2008). We assumed that there was no entry of our target population into the treated crop. Calculations thus considered the potential for individuals living in the vicinity of treated orchards to be exposed via inhalation of pesticide vapour and indirect dermal contact with contaminated surfaces for a period of time following the application.

### Pesticide usage data

Information on the use of plant protection products in the UK is required under EU legislation (EC Regulation 1185/09). Pesticide usage data have been collected systematically since 1965 by the Pesticide Usage Survey carried out by Fera Science Ltd. (formerly Central Science Laboratory, and the Food and Environment Research Agency). Field level data were not stored on relational databases until 1987. Prior to this only summary data from the published reports were stored on a relational database. The survey relies on a stratified random sample of farms to estimate total use, allowing comparability of data over time. For the current investigation, orchard data had been collected on a 4-year rolling basis, i.e., 1987, 1992, 1996, 2000, 2004, 2008, and 2012. Collecting data via personal visits to the farms improves accuracy as surveyors can scrutinise all potential pesticide uses which might have occurred to ensure the farmers do not omit or forget anything important (Thomas 1999; Eurostat 2008).

In this study, we first evaluated changes in usage across all survey years and then selected 4 years for more detailed analysis to estimate changes in exposure and risk to health. The first orchard usage data were collected in 1983, but methodology was not consistent with subsequent studies. Hence, 1987 was chosen as the starting year and 1996, 2004, and 2012 were included to give approximately 8-year intervals up to the latest survey reported at the time of analysis. The main orchard crops grown in England and Wales are listed in Table 1 alongside the four regions of England and Wales included in the analysis on the basis that together they accounted for 95.8% of total orchard cultivation in 2012 (Fig. S1). A total of 132 individual active substances are identified within the usage surveys as having been applied to major orchard crops in at least one of the years considered. The application rate, *AR* of an active substance for every application was one of the major factors in the exposure modelling. We estimated the average rate applied to each hectare of orchard from statistics for total amount applied and total area of each crop grown in a region. We calculated the exposure from applications of individual active substances based on monthly usage statistics. Hence, both treatments with a single substance in successive months or a single treatment with a product containing two active substances would both count as two applications in the exposure calculation.

**Table 1:** Area of major orchard crops in four regions that accounted for 95.8% of total orchard cultivation in England and Wales in 2012 (Garthwaite et al. 2012)

Crop type	Crop area grown (ha)				
	Eastern	West Midlands	South-Eastern	South-Western	Total for England and Wales
Cherries	27	187	464	1	697
Cider apples/perry pears	83	5244	41	2731	8619
Culinary apples (Bramley)	585	47	1438	10	2140
Culinary apples (others)	129	–	1	8	146
Dessert apples (Cox)	277	288	1317	33	1960
Dessert apples (others)	419	414	3367	86	4447
Other top fruit (incl. nuts)	45	–	131	36	213
Pears	340	88	1295	24	1757
Plums	160	170	426	150	973
Total grown area	2065	6438	8480	3079	20,952
% of total area	9.9	30.7	40.5	14.7	100.0

### Models for pesticide fate and exposure

Exposure calculations predicted the maximum daily exposure (mg kg bw^−1^ day^−1^) to each active substance applied to orchard crops, calculating the exposure as that for the first 24 h after pesticide application. The EFSA assessment for residents’ exposure to pesticides is currently based on the highest time-weighted average exposure for the first 24 h after application via inhalation from vapour and 2 h of dermal exposure to surface deposits (EFSA 2014). The FOCUS Air group considered that the largest exposure would occur within a 24-h period following application when taking into account the effects of dilution and dispersion of residues due to changing meteorological conditions (FOCUS 2008). Here, we used a simplified additive method to calculate the exposure to, and the cumulative reproductive and/or developmental risk associated with, all pesticides applied to a single orchard crop type across a chosen year. Dissipation of active substances in soil and on plant surfaces was not included, so no attempt was made to estimate the change in exposure during the days/weeks after treatment.

A new model was developed to estimate exposure via inhalation of vapour, drawing on existing algorithms used in PEARL (Pesticide Emission Assessment at Regional and Local scales; van den Berg and Leistra 2004), PELMO (Pesticide Leaching Model; Ferrari et al. 2005), and ISCST2 (Industrial Source Complex Short Term 2; US EPA 1992a). Indirect dermal contact with contaminated ground was estimated from the equations provided by EFSA (2014) for systemic exposures of residents via dermal routes. Where parameters were set to default values, these are listed in Table S1.

#### Volatilisation from treated surfaces (source emission)

Algorithms from the PEARL and PELMO models were adjusted to estimate the rate of pesticide emissions after application from plant and soil surfaces, respectively. The PEARL model incorporates the concept of atmospheric resistance to pesticide volatilisation based on the thickness of laminar air boundary layers and diffusion of vapour from the plant surface to the turbulent air. It incorporates the effect of prevailing meteorological conditions on the initial estimation of pesticide volatilisation from crops in the field. PELMO estimates volatilisation from soil water by assuming negligibly low concentration of pesticide in the air above the soil (not including soil-air partitioning) (Wolters et al. 2003). Other competing processes for dissipation of pesticides in different environmental compartments were not included in our calculations so that leaching, transformation and wash-off from plant surfaces were all excluded, creating a more protective risk assessment.

The saturated vapour concentration of pesticide in the gas phase at the plant surface, ${C}_{g,{p}_s}$ (g m^−3^), depends on its substance-specific vapour pressure at the prevailing temperature. ${C}_{g,{p}_s}$ is calculated using the Gas Law as described by van den Berg and Leistra (2004):

1 $$ {\boldsymbol{C}}_{\boldsymbol{g},{\boldsymbol{p}}_{\boldsymbol{s}}}=\frac{\boldsymbol{M}\bullet \boldsymbol{VP}\left(\boldsymbol{T}\right)}{\boldsymbol{R}\bullet \boldsymbol{T}} $$

where *M* is the molecular mass (g mol^−1^), *VP*(*T*) is the vapour pressure of the pesticide (Pa) as a function of temperature based on PPDB (2017), *R* is the universal gas constant (Pa m^3^ K^−1^ mol^−1^), and *T* is the air temperature (K). The potential rate of volatilisation of pesticide from the leaf surface, *J*_*plant*_ (g m^−2^ day^−1^) is calculated as:

2 $$ {\boldsymbol{J}}_{\boldsymbol{v},\boldsymbol{pot}}\kern0.5em =\frac{{\boldsymbol{C}}_{\boldsymbol{g},{\boldsymbol{p}}_{\boldsymbol{s}}}-{\boldsymbol{C}}_{\boldsymbol{air}}}{\boldsymbol{r}} $$

where *C*_*air*_ is the concentration in the turbulent air just outside the laminar air layer (g m^−3^), and *r* is the resistance to transport from plant surface to atmosphere (d m^−1^) calculated as the ratio of thickness of the boundary air layer, *d* (m) to the adjusted air diffusion coefficient, *D*_*a*_ (m^2^ day^−1^). It has been proposed that *d* ranges between 0.05 and 0.1 cm depending on the micrometeorological conditions (e.g. air velocity and turbulence) and surface properties (e.g. temperature and roughness) (Leistra and Wolters 2004; FOCUS 2008; Lichiheb et al. 2014; Houbraken et al. 2016). We used default values of 0.06 and 0.1 cm for the thickness of the boundary air layers on plant leaves and soil surfaces, respectively (van den Berg et al. 2016); sensitivity of rate of pesticide volatilisation to the value of *d* (Fig. S2) illustrates the inversely proportional relationship (a doubling in *d* halves the emission rate). However, all the areic quantities such as fluxes are expressed per m^2^ field surface (not plant surface). Consequently, the actual rate of pesticide volatilisation from plant surfaces, *J*_*plant*_ (g m^−2^ day^−1^; maximum daily emission is the mass of pesticide per unit area of plant immediately after application) is estimated by taking into account the mass of pesticide on the plants:

3 $$ {\boldsymbol{J}}_{\boldsymbol{plant}}={\boldsymbol{f}}_{\boldsymbol{mas}}\bullet {\boldsymbol{J}}_{\boldsymbol{v},\boldsymbol{pot}} $$

where *f*_*mas*_ (dimensionless) is the factor to adjust amount of pesticide present on the plants as described by:

4 $$ {\boldsymbol{f}}_{\boldsymbol{mas}}=\frac{{\boldsymbol{A}}_{\boldsymbol{p}}}{{\boldsymbol{A}}_{\boldsymbol{p},\boldsymbol{ref}}} $$

where *A*_*p*_ refers to the areic mass of pesticide on the plants (g m^−2^) obtained by multiplying application rate, *AR* (g m^−2^) with the crop interception factor, and *A*_*p* , *ref*_ is the reference areic mass of pesticide on the plants. This assumes that thinner deposits on the leaves will be depleted sooner and the volatilising surface decreases along with the mass of pesticide in the deposit.

Algorithms from PELMO were used in the estimation of pesticide emission rates from exposed soil surfaces on a daily basis (Wolters et al. 2003; Ferrari et al. 2005):

5 $$ {\boldsymbol{J}}_{\boldsymbol{soil}}\kern0.5em =\frac{{\boldsymbol{H}}^{\boldsymbol{\hbox{'}}}{\boldsymbol{c}}_{\boldsymbol{sol}}}{\boldsymbol{r}} $$

where *J*_*soil*_ is the volatilisation rate from soil (g m^−2^ day^−1^; maximum daily emission is the mass of pesticide per unit area of soil immediately after application), *D*_*a*_ is the diffusion coefficient in air (m^2^ day^−1^), *H* ^'^is the non-dimensional Henry’s law constant, *d* is the air boundary layer (m), *c*_*sol*_ is pesticide concentration in the soil pore water (g cm^−3^), and *r* is the resistance to transport from the soil surface to the atmosphere as calculated in Eq. 2 (d m^−1^). Adjustments were required for three temperature-dependent parameters, namely *D*_*a*_, *H* ^'^ and *VP*, whilst *c*_*sol*_ depends on application rate and the substance-specific organic carbon partition coefficient, *K*_*oc*_ (mL g^−1^), with the use of default values for fraction of organic carbon, *f*_*oc*_, soil water content (g g^-1^), and dry soil bulk density (g cm^−3^). According to Leistra et al. (2001), *D*_*a*_ was adjusted with:

6 $$ {\boldsymbol{D}}_{\boldsymbol{a}}={\boldsymbol{D}}_{\boldsymbol{a},\boldsymbol{ref}}\ {\left(\frac{\boldsymbol{T}}{{\boldsymbol{T}}_{\boldsymbol{ref}}}\right)}^{1.75} $$

where *D*_*a* , *ref*_ is the diffusion coefficient in air at 20 °C, and *T*_*ref*_ is the reference temperature at 20 °C. *H* ^'^ was adjusted with a *Q* *10* factor that was derived as the median value of a range of factors (1.15–2.28) that have been reported for different active substances (Staudinger and Roberts 2001; Feigenbrugel et al. 2004; Cetin et al. 2006). *Q* *10* is defined as the ratio of degradation rates between the rates at 20° and 10 °C (EFSA 2007). According to Sarigiannis et al. (2013),

7 $$ \boldsymbol{VP}={\boldsymbol{VP}}_{\boldsymbol{ref}}\cdot \boldsymbol{\exp}\ \left[-\frac{\varDelta {\boldsymbol{H}}_{\boldsymbol{vap}}}{\boldsymbol{R}}\ \left(\frac{1}{\boldsymbol{T}}-\frac{1}{{\boldsymbol{T}}_{\boldsymbol{ref}}}\right)\right] $$

where *VP*_*ref*_ is the saturated vapour pressure of the substance at reference conditions (mPa), *ΔH*_*vap*_ is the molar enthalpy of evaporation (J mol^−1^), *R* is the universal gas constant (J K^−1^ mol^−1^), *T* is the air temperature (K), and *T*_*ref*_ is the reference air temperature (K).

Two parameters were shared between calculations for volatilisation from the two surfaces, namely the crop interception factor (*CI*) and monthly air temperature. For *CI*, emission rates of the pesticide from treated surfaces (plant and soil) were both estimated based on pesticide deposition at different growth stages (Leistra et al. 2001). *CI* values for apple trees were obtained from FOCUS (2000) and applied in calculations for all other orchard crops (Table S2). The proportion of sprayed pesticide reaching the soil surface was calculated by difference. Mean monthly air temperatures for the past 35 years (1980–2015) were obtained from the Meteorological Office as regional climatic records and the 35 values for each month were averaged to derive monthly air temperature values to input into the calculations (Table S3).

The area source emission rate (*Q*_*act*_, g m^−2^ s^−1^) from all treated surfaces was calculated for each application of an active substance:

8 $$ {\boldsymbol{Q}}_{\boldsymbol{act}}=\frac{\left({\boldsymbol{J}}_{\boldsymbol{plant}}+{\boldsymbol{J}}_{\boldsymbol{soil}}\right)}{86,400} $$

where 86,400 converts the units of time from days to seconds.

#### Dispersion of volatilised pesticides downwind

A Gaussian diffusion model was used to estimate airborne concentrations of pesticide at different distances downwind of the emission source. ISCST2 was chosen because it is adaptable to various types of source emissions (i.e. point sources, volume sources, and area sources). The area source model of ISCST2 has frequently been used to assess the effects of pollutants on local air quality using emission rates and meteorological conditions as model inputs (Abdul-Wahab 2004). It is adjustable for various parameters including height of crops (m), treated area (ha), wind speed (m s^−1^), and mixing height (m).

By assuming that no crosswind (*y*=0) occurs at the area source and that atmospheric conditions are neutral, the total emission rate from both soil and plant surfaces was translated into airborne pesticide concentration at downwind distance, *X* (m) (measured from the downwind edge of the source area) by:

9 $$ \boldsymbol{X}=\frac{{\boldsymbol{Q}}_{\boldsymbol{act}}\cdot \boldsymbol{V}\cdot \boldsymbol{E}\cdot {\boldsymbol{X}}_{\boldsymbol{o}}}{4\cdot \sqrt{2}\cdot {\boldsymbol{U}}_{\boldsymbol{s}}\cdot {\boldsymbol{\sigma}}_{\boldsymbol{z}}} $$

where *Q*_*act*_ is the area source emission rate (g m^−2^ s^−1^), *V* is the vertical term (−), *E* is the error function term (−), *X*_*o*_ is the length of the side of the square area source (m), *U*_*s*_ is the wind speed (m s^−1^), and *σ*_*z*_ is the vertical standard deviation (−).

The parameter, *V* was required to change the form of the vertical concentration distribution from Gaussian to rectangular (uniform concentration within the surface mixing layer) at downwind distances as follows:

10 $$ \boldsymbol{V}=\boldsymbol{\exp}\left[-0.5{\left(\frac{{\boldsymbol{z}}_{\boldsymbol{r}}-{\boldsymbol{h}}_{\boldsymbol{e}}}{{\boldsymbol{\sigma}}_{\boldsymbol{z}}}\right)}^2\right]+\boldsymbol{\exp}\left[-0.5{\left(\frac{{\boldsymbol{z}}_{\boldsymbol{r}}+{\boldsymbol{h}}_{\boldsymbol{e}}}{{\boldsymbol{\sigma}}_{\boldsymbol{z}}}\right)}^2\right]+\sum_{\boldsymbol{i}=1}^{\infty}\left\{\boldsymbol{\exp}\left[-0.5\ {\left(\frac{{\boldsymbol{z}}_{\boldsymbol{r}}\hbox{--} \left(2{\boldsymbol{i}\boldsymbol{z}}_{\boldsymbol{i}}\hbox{--} \boldsymbol{he}\right)}{{\boldsymbol{\sigma}}_{\boldsymbol{z}}}\right)}^2\right]+\boldsymbol{\exp}\left[-0.5{\left(\frac{{\boldsymbol{z}}_{\boldsymbol{r}}+\left(2{\boldsymbol{i}\boldsymbol{z}}_{\boldsymbol{i}}\hbox{--} \boldsymbol{he}\right)}{{\boldsymbol{\sigma}}_{\boldsymbol{z}}}\right)}^2\right]+\boldsymbol{\exp}\left[-0.5{\left(\frac{{\boldsymbol{z}}_{\boldsymbol{r}}\hbox{--} \left(2{\boldsymbol{i}\boldsymbol{z}}_{\boldsymbol{i}}+\boldsymbol{he}\right)}{{\boldsymbol{\sigma}}_{\boldsymbol{z}}}\right)}^2\right]+\boldsymbol{\exp}\left[-0.5{\left(\frac{{\boldsymbol{z}}_{\boldsymbol{r}}+\left(2{\boldsymbol{i}\boldsymbol{z}}_{\boldsymbol{i}}+\boldsymbol{he}\right)}{{\boldsymbol{\sigma}}_{\boldsymbol{z}}}\right)}^2\right]\right\} $$

where *h*_*e*_ is the crop height (m), *z*_*r*_ is adult height above ground (m), and *z*_*i*_ is the mixing height (m) adjusted based on crop height (Randerson 1984) with:

11 $$ {\boldsymbol{z}}_{\boldsymbol{i}}=\frac{0.3\ {\boldsymbol{u}}^{\ast}}{\boldsymbol{f}} $$

where *f* is the Coriolis parameter (s^−1^ at 40° latitude) and *u* ^∗^ is friction velocity (m s^−1^) calculated for the reference wind speed, *u*(*z*) at 2.0 m above the ground using the logarithmic wind profile relationship:

12 $$ \boldsymbol{u}\left(\boldsymbol{z}\right)=\frac{{\boldsymbol{u}}_{\ast}}{\boldsymbol{k}}\boldsymbol{In}\ \left(\frac{\boldsymbol{z}}{{\boldsymbol{z}}_0}\right) $$

where *k* is the von Karman’s constant (dimensionless) and *z*_0_ is the roughness parameter (m) approximated as 10% of the height of the crop surface.

The error function term, *E* is described by:

13 $$ \boldsymbol{E}=\boldsymbol{\operatorname{erf}}\left(\ \frac{{\boldsymbol{r}}_{\boldsymbol{o}}^{\boldsymbol{\hbox{'}}}+\boldsymbol{y}}{\sqrt{2}{\boldsymbol{\sigma}}_{\boldsymbol{y}}}\right)+\boldsymbol{\operatorname{erf}}\left(\frac{{\boldsymbol{r}}_{\boldsymbol{o}}^{\boldsymbol{\hbox{'}}}-\boldsymbol{y}}{\sqrt{2}{\boldsymbol{\sigma}}_{\boldsymbol{y}}}\right) $$

where *r*_*o*_^'^ is the effective radius of area source $\frac{X_o}{\surd \pi }$ (m), and σ_y_ is the lateral vertical standard deviation.

The dispersion parameters were calculated according to a power-law fit to wind tunnel data (US EPA 1992b):

14 $$ {\boldsymbol{\sigma}}_{\boldsymbol{y}}=0.73547\ {\boldsymbol{X}}^{0.64931} $$

15 $$ {\boldsymbol{\sigma}}_{\boldsymbol{z}}=0.28565\ {\boldsymbol{X}}^{0.71285} $$

#### Calculation of inhalation exposure

Concentrations in air derived from the air dispersion modelling were converted into individual exposures according to EFSA (2014):

16 $$ {\boldsymbol{SER}}_{\boldsymbol{I}}=\frac{\boldsymbol{VC}\cdot \boldsymbol{IR}\cdot \boldsymbol{A}}{\boldsymbol{BW}} $$

where *SER*_*I*_ is defined as the systemic exposure of residents via the inhalation route (mg kg bw^−1^ day^−1^), *VC* is the estimated pesticide vapour concentration (mg m^−3^) at the selected proximity, *IR* is inhalation rate (m^3^ day^−1^), *IA* is inhalation absorption (−), and *BW* is body weight (kg).

Inhalation rate was set to 13.8 m^3^ day^−1^ based on default values for an adult female of 0.23 m^3^ day^−1^ kg^−1^ daily inhalation rate of residents to vapours and 60 kg body weight for adults (US EPA 2009; EFSA 2010). A literature search was undertaken for information on absorption factors via the lungs following inhalation of pesticides; there is no consistent information on this process, so a default value of 100% absorption via inhalation was used (Butler Ellis et al. 2013; EFSA 2014; GroBkopf et al. 2013). Body weight for an adult female was set to 60 kg as recommended by EFSA (2014).

#### Calculation of indirect dermal exposure

Systemic exposure via the dermal route, *SER*_*D*_ (mg kg bw^−1^ day^−1^) was calculated according to EFSA (2014):

17 $$ {\boldsymbol{SER}}_{\boldsymbol{D}}=\frac{\boldsymbol{AR}\cdot \boldsymbol{D}\cdot \boldsymbol{TTR}\cdot \boldsymbol{TC}\cdot \boldsymbol{H}\cdot \boldsymbol{D}\boldsymbol{A}}{\boldsymbol{BW}} $$

where *AR* is the application rate (mg cm^−2^), *TTR* is the turf transferable residue (−), *TC* is the transfer coefficient (cm^2^ h^−1^), *H* is the exposure duration (hour), *DA* is the dermal absorption (−), and *BW* is the body weight (kg). *D* is the drift fraction which is calculated in accordance with crop growth stages:

18 $$ \mathbf{For}\ \mathbf{early}\mathbf{growth}\mathbf{stages},\boldsymbol{D}=\left(\frac{3908.3^{\ast}\left({\boldsymbol{X}}^{-2.421}\right)}{100}\right) $$

19 $$ \mathbf{For}\ \mathbf{late}\mathbf{growth}\mathbf{stages},\boldsymbol{D}=\left(\frac{298.83^{\ast}\left({\boldsymbol{X}}^{-1.8672}\right)}{100}\right) $$

20 $$ \mathbf{For}\ \mathbf{downward}\mathbf{herbicide}\mathbf{applications},\boldsymbol{D}={2.7705}^{\ast}\left({\boldsymbol{X}}^{-0.9787}\right) $$

where *X* is the selected downwind distance (m) (Rautmann et al. 1999).

Dermal absorption (DA) values for individual active substances (*n* = 132) were extracted from the EFSA scientific reports on peer review of risk assessments for individual active substances, EFSA DAR and the Risk Characterisation Documents from the California Department of Pesticide Regulation; a default value of 75% was used for substances where no measured values were found (EFSA 2012).

#### Calculation of total exposure

Estimated levels of exposure (mg kg bw^−1^ day^−1^) to individual active substances for the two identified routes/pathways were summed to give an aggregated exposure:

21 $$ {\boldsymbol{\varSigma} \boldsymbol{Exposure}}_{\left(\boldsymbol{AS}\right)}={\boldsymbol{Exposure}}_{\left(\boldsymbol{Inhaled}\boldsymbol{vapour}\right)}+{\boldsymbol{Exposure}}_{\Big(\boldsymbol{indirect}\boldsymbol{dermal}}\Big) $$

Subsequently, the total exposures to individual substances were summed to give an aggregated exposure for individual crops:

22 $$ {\boldsymbol{\varSigma} \boldsymbol{Exposure}}_{\left(\boldsymbol{crop}\boldsymbol{type}\right)}={\boldsymbol{Exposure}}_{\left({\boldsymbol{AS}}_{\boldsymbol{i}}\right)}+\dots +{\boldsymbol{Exposure}}_{\left({\boldsymbol{AS}}_{\boldsymbol{i}+\boldsymbol{n}}\right)} $$

Timing of exposure to different compounds was not explicitly considered in the calculation and is discussed as a constraint on the methodology in “Discussion” section.

### Risk estimation

Generally, regulatory risk assessment of pesticides in the EU is undertaken for single active substances or single pesticide products (Stehle and Schulz 2015). The implementation of cumulative and combined exposures to pesticides is explicitly required by the regulatory agencies under Regulation (EC) 1107/2009 (Stein et al. 2014; Panizzi et al. 2017). The use of dose addition in regulatory risk assessment is considered sufficiently conservative as a default first tier approach for cumulative assessment, where the risk is deemed acceptable if the sum of all hazard quotients (HQ) ≤ 1 (Sarigiannis and Hansen 2012; Stein et al. 2014). The risk from exposure to individual active substances was calculated based on the hazard quotient (HQ) approach:

23 $$ \boldsymbol{HQ}=\frac{\boldsymbol{Exposureestimate}\ \boldsymbol{for}\ \boldsymbol{individual}\ \boldsymbol{AS}\ }{\boldsymbol{Referencepoint}\ } $$

The reference point in this research refers to the no observed (adverse) effect level (NO(A)EL) for reproductive and/or developmental effects for individual substances. Reference points were extracted from four established toxicological databases, namely the EFSA Draft Risk Assessment Report (DAR) and Assessment Report (AR) (http://dar.efsa.europa.eu/dar-web/provision), the Joint Meeting on Pesticide Residues (JMPR) of the International Programme on Chemical Safety (IPCS INCHEM, http://www.inchem.org/pages/jmpr.html), the Integrated Risk Information System (IRIS, https://www.epa.gov/iris), and the Hazardous Substances Data Bank (HSDB) in the Toxicology Data Network (TOXNET, https://toxnet.nlm.nih.gov/newtoxnet/hsdb.htm).

One of the major issues in selecting the most relevant threshold for an individual active substance was the unclear boundary between reproductive and developmental effects for different periods of exposure (i.e. before pregnancy and during different trimesters). For instance, the EFSA DAR defines reproductive toxicities based on endpoints such as reduced offspring body weight or liver weight in two- and/or three-generation studies whilst developmental toxicities are assessed based on endpoints such as skeletal malformation, teratogenicity, and foetotoxicity. Meanwhile, the JMPR interprets the reproductive parameters as number of implants, resorptions, and dead foetuses, and developmental parameters refers to post-implantation variation in foetuses, and decreased viability indices. Generally, reproductive toxicity refers to any toxicological effects that may occur at different phases within the reproductive cycle whilst developmental toxicity refers to any effects in prenatal developmental studies and in one- or multi-generation studies (Wolterink et al. 2013). Since the test parameters were not uniquely classified, the lowest NO(A)ELs for reproductive and/or developmental effects were selected for use. As for the different thresholds in four different toxicological databases due to different study designs, the lowest NO(A)ELs for either reproductive or developmental toxicity were selected for use. This approach avoids any exclusion of potential higher toxicity for an individual active substance. It was found that 8 out of the 132 active substances applied to orchards in our dataset have no published toxicological thresholds for reproductive and/or developmental effects due to their chemical structure and here no NO(A)ELs was allocated (Table S4). For four active substances with significant use in at least one of the study years, the NO(A)EL were allocated based on either a major constituent in the compound (benzo-a-pyrene for tar oil), or similarity of chemical structures (dichlorprop-P/dichlorprop and mecoprop-P/mecoprop). Heptenophos has no data but is expected to be hazardous, so the NOAEL for chlorpyrifos was used, whilst the NOAEL for metiram was estimated by dividing the published LOAEL by two.

Studies on inhalation toxicity are lacking for most pesticides. Approximately 80% of inhalation risk assessments are based on route-extrapolated oral studies, whilst 20% of inhalation NOAEL data are route-extrapolated to dose (in mg kg bw^−1^ day^−1^) from measured air concentrations (Salem and Katz 2006). In the absence of data, the inhalation NOAEL is typically extrapolated from an oral study by assuming inhalation absorption is 100% of oral absorption due to the likelihood of higher absorbed dose via the inhalation route (Kegley and Conlisk 2010).

## Results

### Pesticide usage

**Fig. 1:**
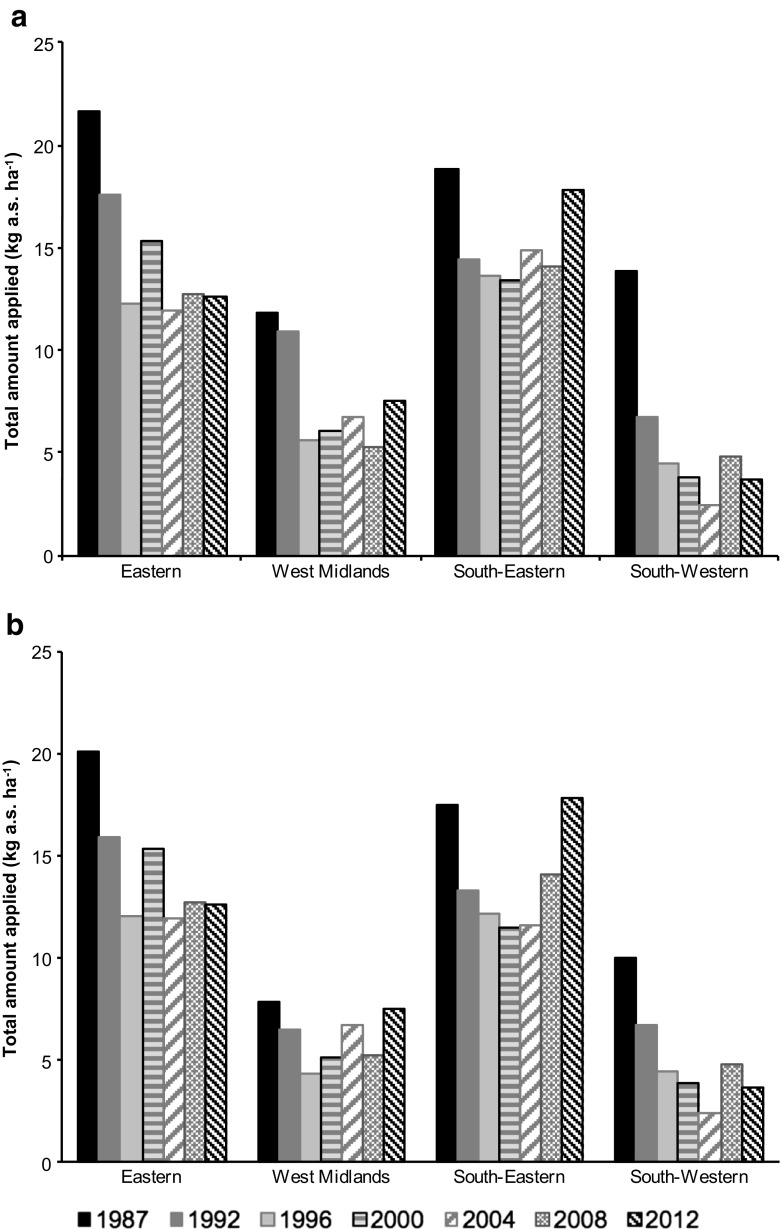
Changes between 1987 and 2012 in total amount of pesticide applied to orchards cultivated in four regions of England and Wales. Data are shown either with tar oils included (**a**) or excluded (**b**)

Figure 1 shows changes in total amount of pesticides applied to orchards in the four regions over a 25-year period with 4-year intervals. Data are shown with (Fig. 1a) and without (Fig. 1b) applications of tar oils as some of the associated rates of application were large and could mask changes in the other active substances used. Across the full period, the total amount of pesticide applied in any 1 year ranged between 2.0 and 21.0 kg ha^−1^. Generally, there was greater usage of pesticide for orchards in the Eastern and South-Eastern regions compared to the West Midlands and South-Western regions. The total amount of pesticide applied was always greatest in 1987 and had decreased by 1992 and 1996 in all four regions. In contrast, no consistent changes were found for the later survey years (1996–2012) with some increases in total amounts applied in specific years between 2000 and 2012. The results revealed that the South-Western region had a large decrease in total applied amounts from 1987 to 1992, followed by a constant decline from 1992 to 2004 and inconsistent changes between 2004 and 2012. In contrast, total pesticide used in the South-Eastern region was approximately equal in 1987 and 2012 independent of whether or not tar oils were included.

The results were further analysed for four chosen years with approximately 8-year intervals from 1987 up to 2012 to investigate trends in pesticide usage for individual crop types. Tar oils were excluded from this analysis as they significantly skewed the total application amounts for plums and cherries in 1987 and to a lesser extent in 1996 and 2004. For instance, the highest application rate for plums in the South-Western region in 1987 (60.2 kg a.s. ha^−1^) and cherries in the West Midlands region in 1987 (35.6 kg a.s. ha^−1^) comprised 98.6 and 99.8% tar oils, respectively (Fig. S3).

**Fig. 2:**
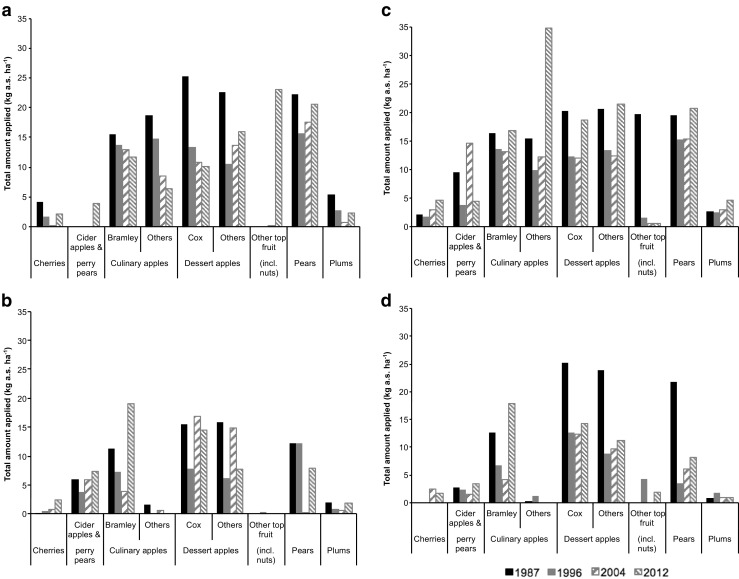
Total amount of pesticide applied to major orchard crop types between 1987 and 2012 for Eastern (**a**), West Midlands (**b**), South-Eastern (**c**), and South-Western (**d**) regions. Blanks indicate that none of that orchard type was sampled in that region and tar oils are excluded from the data as large application rates obscure other trends

**Fig. 3:**
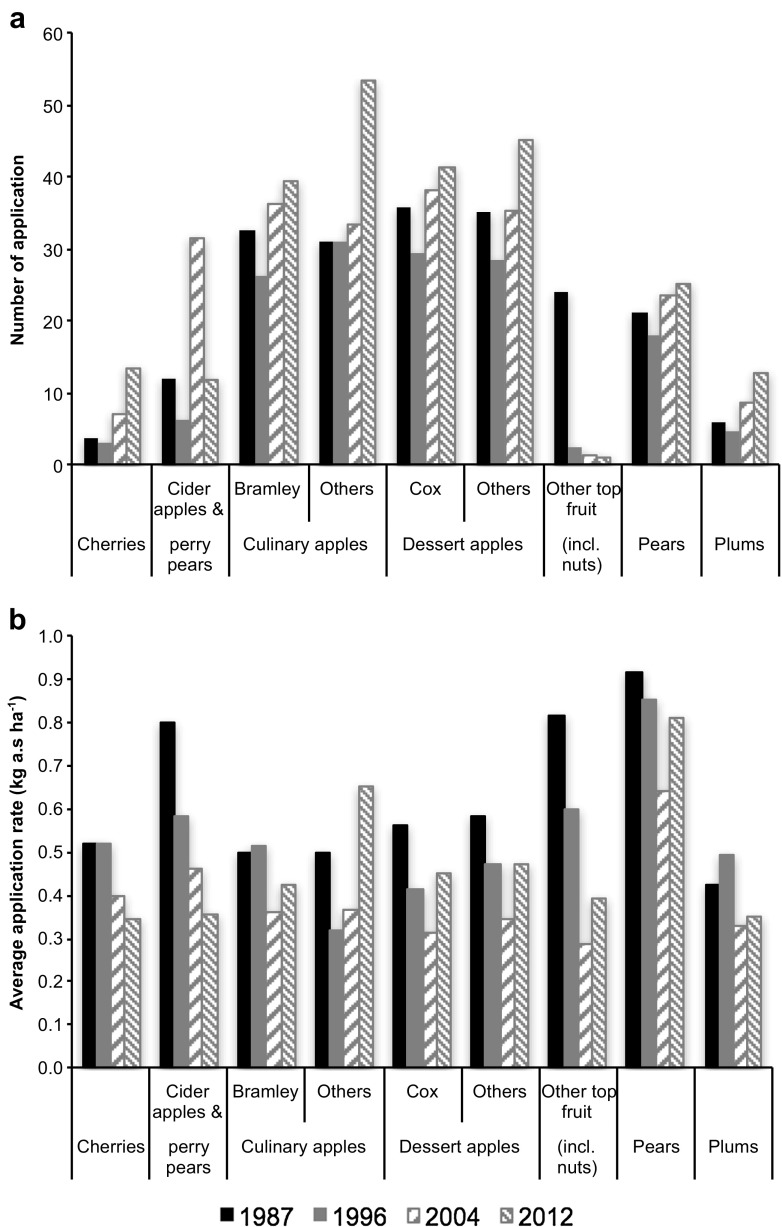
Usage of pesticide for orchard crop types cultivated in the South-Eastern region with usage of tar oils excluded. Data are expressed as number of applications (**a**) defined as treated area divided by area grown, and average application rate (**b**) defined as total amount applied divided by number of applications. Here, application is defined as one treatment with one active substance, so successive treatments with a single active substance or a single treatment with a product containing two active substances would both count as two applications

Total amount of pesticides applied to individual crop types was generally less than 30.0 kg a.s. ha^−1^ when tar oils were excluded (Fig. 2). Some consistently low application amounts were identified for crops such as cherries, other top fruit and plums in all four regions (Fig. 3b; Fig. S4b) although sample size was small due to the small area of each crop grown. The Eastern region showed declining trends of total application amounts for culinary apples (Bramley and others) and dessert apples (Cox) from 1987 to 2012. Meanwhile, the West Midlands and South-Western regions with relatively smaller pesticide usage showed no significant trends. Most crop types in the South-Eastern region had higher total application amounts in 2012 as compared to 2004. When tar oils were removed from the dataset, the greatest total amount of pesticide applied was for culinary apples (others) in the South-Eastern region in 2012 that comprised 71.5% captan, 8.3% chlorpyrifos, 6.0% dithianon, and 14.2% other substances.

Figure 3 presents the usage data as total number of applications of an active substance and as average rate of application across all treatments. There has generally been an increase in the number of applications of an active substance (Fig. 3a), but this has been accompanied by a general decrease in the average rate of application (Fig. 3b). The average application rate (Fig. 3b) better explains the trends in pesticide usage with similar patterns to those shown in Fig. 1, i.e. the highest average application rates and total applied amounts were in 1987 for all chosen regions (Fig. S4).

### Aggregated exposures for residents living 100 m downwind

**Fig. 4:**
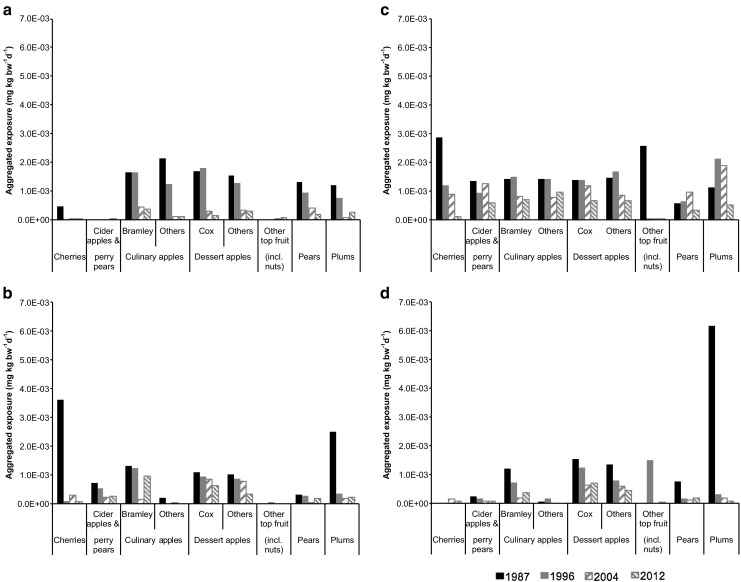
Aggregated exposures to applied pesticide for residents living 100 m downwind of individual crop types. Data are shown for 4 years between 1987 and 2012 and for Eastern (**a**), West Midlands (**b**), South-Eastern (**c**), and South-Western (**d**) regions

Aggregated exposure to pesticides via inhaled pesticide vapour and contact with contaminated ground were estimated for residents living 100 m downwind of individual crop types. Tar oils were included in all estimations of exposure and risk. Aggregated exposures to individual crop types were generally smaller than 2.0 × 10^−3^ mg kg bw^−1^ day^−1^ with most of the largest estimates in 1987 and values decreasing over the survey years (Fig. 4). The Eastern and South-Western regions showed decreasing trends for most of the crop types whilst the West Midlands region showed less consistency in aggregated exposures. In comparison, the South-Eastern region indicated relatively high and constant exposures with small changes over the years. Overall, the exposures were smallest in 2012 with a couple of exceptions including culinary apples (Bramley) in the West Midlands region that increased approximately sevenfold from 2004 (1.4 × 10^−4^ mg kg bw^−1^ day^−1^) to 2012 (9.6 × 10^−4^ mg kg bw^−1^ day^−1^). In some cases, aggregated exposures greater than 2.0 × 10^−3^ mg kg bw^−1^ day^−1^ were strongly affected by tar oils, i.e. plums in the South-Western region in 1987 (6.1 × 10^−3^ mg kg bw^−1^ day^−1^) and cherries in the West Midlands region in 1987 (3.6 × 10^−3^ mg kg bw^−1^ day^−1^) where total exposure was approximately 99.5% attributable to tar oils.

### Aggregated hazard quotients for residents living 100 m downwind

**Fig. 5:**
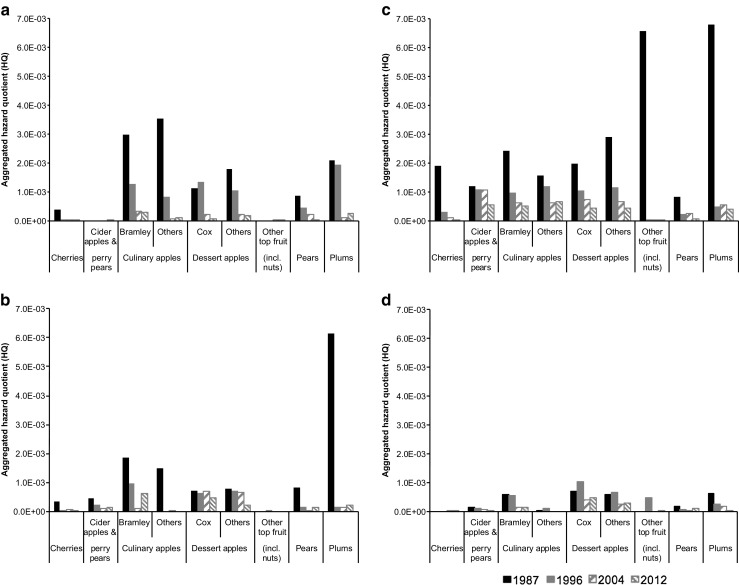
Aggregated hazard quotients of reproductive and/or developmental toxicities to applied pesticide of resident pregnant women living 100 m downwind of individual crop types. Data are shown for four years between 1987 and 2012 and for Eastern (**a**), West Midlands (**b**), South-Eastern (**c**), and South-Western (**d**) regions

Exposure estimates were converted into HQs using reproductive and/or developmental toxicities of the applied pesticides. Figure 5 shows that all aggregated HQs were at least two to three orders of magnitude smaller than 1, despite the inherent simplifications of assuming co-occurrence of exposure to all pesticides and additivity of effects. 1987 had the highest aggregated HQs and these decreased greatly by 1996, followed by smaller changes between 1996 and 2012. Generally, the Eastern, West Midlands, and South-Western regions had relatively lower aggregated HQs for most of the crop types compared to those for the South-Eastern region. Aggregated HQs were smallest in 2012 for most crop types, but with exceptions including culinary apples (Bramley) in the West Midlands region that increased approximately six-fold in 2012 (6.2 × 10^−4^) when compared to 2004 (9.9 × 10^−5^). For individual crop types with relatively larger aggregated HQs, results were influenced significantly by one or two dominant active substances. For instance, the highest aggregated HQ for plums in the South-Eastern region in 1987 (6.8 × 10^−3^) comprised 95.6% demeton-S-methyl and 4.4% other substances; that for 1996 (5.0 × 10^−4^) comprised 47.8% chlorpyrifos, 36.4% tar oil, 7.6% demeton-S-methyl, and 8.2% other substances; that for 2004 (5.5 × 10^−4^) comprised 72.3% chlorpyrifos, 26.0% tar oil, and 1.7% other substances; and that for 2012 (4.1 × 10^−4^) comprised 96.3% chlorpyrifos and 3.7% other substances.

### Aggregated exposures and hazard quotients at 1000 m downwind

Aggregated exposures and risks to health were also estimated for residents living 1000 m from the treated orchard. Aggregated exposures to most of the crop types were smaller than 3.0 × 10^−4^ mg kg bw^−1^ day^−1^ (Fig. S5) with exposure in 1987 and 1996 again estimated to be generally larger than that in 2004 and 2012. The estimations indicated decreasing trends in exposure for most crop types, particularly between 1996 and 2012. The aggregated exposures at 1000 m were converted into corresponding aggregated HQs and the results showed the same trends as at 100 m but with much smaller absolute values (Fig. S6). Overall, the aggregated exposures and HQs at 1000 m for different crop types were approximately 5 to 16 times smaller than the equivalent values at 100 m.

## Discussion

We applied consistent methodologies to compare year-on-year changes in pesticide usage, potential for residential exposure to pesticides, potential risk for reproductive or developmental effects on human health, as well as the major drivers of any changes over the past 30 years in England and Wales. It is important to note that aggregated exposures and risks summed daily values into a single measure even though exposure to different active substances will be widely dispersed in time; thus the data should not be taken as true estimates of daily exposure for direct comparison with daily dose thresholds for toxicity.

Based on four representative regions, average of total pesticide usage across the surveyed years showed a significant decrease from 1987 (66.2 kg a.s. ha^−1^) to 1996 (49.8 kg a.s. ha^−1^), followed by smaller changes through to 2012 (41.7 kg a.s. ha^−1^) (Fig. S7). This finding is supported by a time series analysis of orchard fruit production in Great Britain with a decrease of approximately 22% in the mean usage from 1992 (42,000 kg) to 2008 (33,000 kg) (Cross 2012). Our results show an average 13% increase in total usage in 2012 (41.7 kg a.s. ha^−1^) compared to 2008 due to widespread application of fungicides (Fig. S7; Fig. S8) to control scab and powdery mildew in the wet weather conditions (Garthwaite et al. 2012). Our results are expressed as amount of pesticide applied to 1 ha of crop, so are adjusted for any changes in the area of cultivated orchards over time (Thomas 2003). There was a small but relatively consistent increase in the number of applications of individual active substances to crops; this was offset by a small, but relatively consistent decrease in average application rates over the surveyed years (Fig. 3; Fig. S4). This could reflect an increased uptake of reduced-rate applications at less than the maximum recommended label rate and the introduction of new molecules that are active at lower dose rates (Thomas 2003).

We simplified the estimation of exposure by only considering that part of the dose received within 24 h of the pesticide treatment. This should give a maximum dose when expressed on a daily basis. We further simplified within our aggregation procedure, by summing the daily doses and hazard quotients calculated for each individual treatment, independent of when those treatments occurred. Analysis shows that usage and thus exposure were significantly larger between April and July than for the remainder of the year (Fig. S9). The relative sensitivity for reproductive and/or developmental outcomes of exposure pre-conception or during a specific trimester is unknown (Gonzalez-Alzaga et al. 2015). This is because the critical embryologic period is short and limited to the early stage of gestation before the diagnosis of pregnancy (Castilla et al. 2001). The peak in exposure each year suggests that temporal differentiation in health outcomes would be expected if such outcomes were associated with pesticide use (Li et al. 2014). The CHAMACOS study of associations (95% CI) of proximity to methyl bromide use within a 5-km radius during pregnancy (*n* = 442) showed that the second trimester was a critical period for gestational growth and that exposure was associated with a decrease in means of birth weight (21.4 g), length (0.16 cm) and head circumferences (0.08 cm) (Gemmill et al. 2013). Despite the simplifications in producing aggregated estimates of risk, all values for the aggregated hazard quotient were two to three orders of magnitude or more smaller than one. Overall, this suggests a low level of risk to human health for this situation because co-occurrence of exposure to all pesticides applied to a single crop and additivity of effects from all individual active substances were implicit assumptions that will not hold true.

**Fig. 6:**
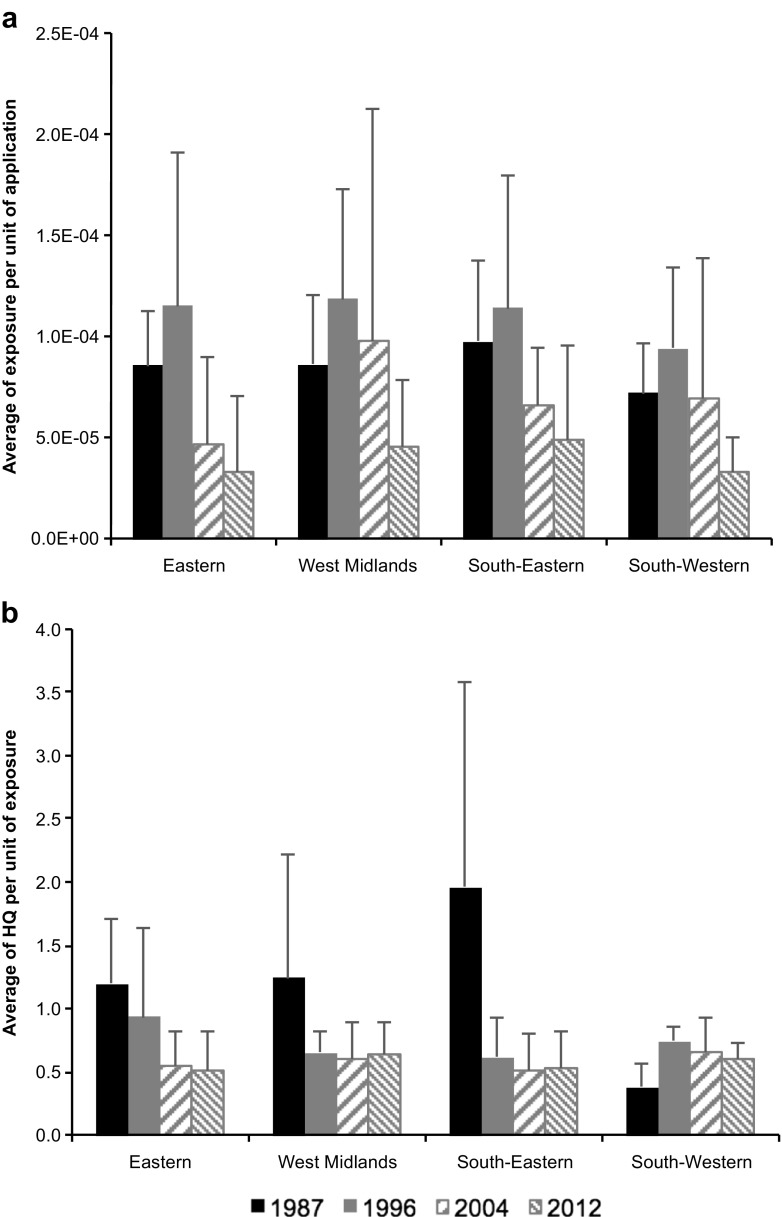
Data for aggregated exposure normalised by expressing per kg of pesticide applied (**a**) and aggregated hazard quotient normalised by expressing per mg kg bw day^−1^ of exposure (**b**). All data are for resident pregnant women living 100 m downwind of treated crops and are shown for 4 years between 1987 and 2012 and for Eastern, West Midlands, South-Eastern, and South-Western regions. Error bars represent standard deviations of exposures and hazard quotients for identified crop types, respectively

Figures 4 and 5 indicate that although there was no consistent change in total pesticide applied to orchard crops over time, there were small decreases in exposure and larger decreases in risk over time for most of the crop and region combinations. To investigate this further, data were normalised to express exposure per unit pesticide applied and risk per unit of exposure (Fig. 6). Overall, there was a small increase in estimated exposure per unit application between 1987 and 1996, but a steady decrease thereafter in all four regions (Fig. 6a). In contrast, there was a relatively large decrease in risk per unit exposure between 1987 and 1996 for three of the four regions, with only small changes thereafter (Fig. 6b). The decrease in risk per unit exposure between 1987 and 1996 can be attributed to the review and withdrawal from the market of compounds with relatively high toxicity for reproductive/developmental effects, including DDT, methidathion, azinphos-methyl, and cyhexatin. This initial impact of deregistrations around the time of introduction of Directive 91/414 is not apparent in the calculations for exposure per unit application (Fig. 6a). However, it is interesting to note that this metric does decrease during the period 1996 to 2012, primarily due to the cessation of use of active substances with relatively higher volatility such as demeton-S-methyl, gamma-HCH, and fenitrothion. Over the full period considered, there has been a clear shift in the properties of pesticides applied to orchards away from compounds with large vapour pressures and small NO(A)ELs (high toxicity) (Fig. S10). FOCUS (2008) proposed a vapour pressure trigger of > 1.0 × 10^−5^ Pa to indicate those substances with potential for significant volatilisation from treated plant surfaces. 61% of the 76 compounds applied to orchards in 1987 had relatively large vapour pressure (> 1.0 × 10^−5^ Pa) and relatively high reproductive/developmental toxicity (NO(A)EL < 10 mg kg bw^−1^ day^−1^); by 2012, this group of substances had reduced to 44% of the 54 compounds applied (Fig. S10). The decreasing trend in total emission rate from treated surfaces and in the resulting concentration in air also indicates the improving fate profile of pesticides applied over the 25-year period (Fig. S11). The sum of airborne concentrations for all pesticides at 100 m decreased by a factor of 3.5 from 1987 (4.6 x 10^-3^ mg m^−3^) to 2012 (1.3 x 10^-3^ mg m^−3^) with concentrations for individual pesticides in the range 4.3 × 10^−17^ to 1.3 × 10^−2^ mg m^−3^. Zivan et al. (2016) measured chlorpyrifos in air collected 74 m downwind from a persimmon orchard in the range 6.3 × 10^−4^ to 2.0 × 10^−3^ mg m^−3^, whilst Coscolla et al. (2010) detected 41 pesticides in ambient air in central France (2006–2008) with individual average concentrations ranging between 1.7 × 10^−7^ mg m^−3^ for vinclozolin and 2.5 × 10^−5^ mg m^−3^ for captan. Overall, the results reflect the influence of changing policies during the 1990s; Cross and Edwards-Jones (2006) found it impossible to identify any single policy leading to changes in pesticide risk over time, but the longer time series analysis possible in our study suggests that the introduction of European Directive 91/414 as well as the ongoing pesticides review programme at national level had a substantive effect in decreasing the overall toxicity profile of pesticides applied to orchards in the UK.

The present study estimated risk of applied pesticides based on maximum aggregated exposure on the first day after the application was made. This is likely to give the maximum daily dose of the pesticide (dose is expressed on a ‘per day’ basis) and indeed some studies show that volatilisation losses of pesticides including chlorpyrifos, prosulfocarb and trifluralin can be nearly complete within 24 h (Rudel 1997; Carlsen et al. 2006; Zivan et al. 2016). Volatilisation of other pesticides including fenpropimorph and parathion-methyl has been shown to proceed over several days or weeks after application (Rudel 1997; Leistra et al. 2008; Kosikowska and Biziuk 2010; Yusa et al. 2014). Whilst the fate of substances beyond the first day after application is not considered in the present work, more prolonged emission of pesticides is possible and could be considered in future studies to provide a more refined assessment of how exposure varies over time. The present work used the hazard quotient as a single figure to assess the risk to human health, combining the toxicity, amount and degree to which humans are exposed (Toronto Public Health 2002). Relatively small exposures were estimated at our selected proximities due to the strong influence of proximity to spraying on magnitude of exposure. Ramaprasad et al. (2009) showed that children of agricultural operators living less than 61 m from an orchard had higher frequencies and greater levels of detectable urinary dimethyl thiophosphate levels than those living farther away. Our results also indicate higher potential hazard for inhalation exposure compared to dermal contact with spray deposits at distances farther downwind from treated orchards. This is due to longer duration of vapour drift because volatilization followed by aerial dispersion generally occurs over longer periods than spray drift and ground deposition (FOCUS 2008). Active substances with greater volatility contributed more to total exposure at 1000 m compared to 100 m; for example, demeton-S-methyl applied to plums in the West Midlands region in 1987 contributed 15.0 and 25.0% of total exposure at 100 and 1000 m, respectively. In contrast, exposure to spray droplets is less likely at greater proximities due to the relatively short time that droplets stay in the air; for example, duration in air is approximately 4 s for fine spray (200 μm in diameter) and 2 s for coarse spray (400 μm) to fall 3 m in still air (Klein et al. 2007).

Several limitations in data availability were encountered during the study. Atmospheric dispersion was the most significant transport pathways for volatilised pesticides yet it is poorly studied with most research focusing on measurements of downwind deposition of pesticide rather than airborne concentrations (Butler Ellis et al. 2010; Zivan et al. 2016). Lack of data on airborne pesticide concentrations and spray deposition at different proximities from treated orchards has been noted previously as a constraint on model validation (Butler Ellis et al. 2013). Our exposure estimates assume that residents receive 24 h of exposure via inhalation of pesticide vapour and 2 h of dermal exposure through activities on the contaminated ground; there is no consideration of structures that might interrupt pathways of exposure such as tree windbreaks, hedges, fences, or houses. We only considered toxicity for reproductive and/or developmental endpoints and did not consider all toxic mechanisms to assess overall potential for impact on health of residents. We also ignored some additional pathways of exposure such as dietary intake because these were assessed as relatively insignificant in the initial problem definition phase. Set against this, we summed daily exposures to all pesticides into a single aggregated value for exposure, even though these exposures will actually be widely spaced in time.

## Conclusion

This study investigated trends in pesticide usage, exposure to pesticides via inhaled vapour and dermal contact with contaminated ground, and risk posed by pesticides applied to orchards for resident pregnant women living 100 or 1000 m downwind of treated areas. The exposure model is flexible and can be adjusted for a range of physicochemical properties of pesticides and atmospheric dispersion parameters. The model should be further validated and improved as field data become available for deposition and airborne concentrations of pesticides at greater distances from the site of application. The explicit calculation of exposures and the long time series of analysis add to the existing body of knowledge and allow a holistic assessment of the impact of pesticide regulation on use, exposure and risk. It is found that quantitative estimation of exposure can express the causal relationship between usage and associated risk in terms of space and time, which is a common caveat in post-authorisation monitoring and epidemiological investigations. There has not been a consistent change in usage over time, with a small increase in number of applications compensated by a small reduction in the average rate applied. Risk levels are generally small and have declined over time, with the cessation of use of several active substances with relatively high toxicity, and a net change to active substances with lower volatility. This evaluation of changes in pesticide use, exposure and risk over a 25-year time span can inform public debate about the effectiveness of regulatory interventions.

## Electronic supplementary material

ESM 1 (PDF 742 kb).
