# Supplementary material for: How does exposure to pesticides vary in space and time for residents living near to treated orchards?
Source: Environ Sci Pollut Res Int. 2017 Sep 25;24(34):26444–61. doi: 10.1007/s11356-017-0064-5 (PMC5719809; doi:10.1007/s11356-017-0064-5)
Supplement: Supplementary file 1 — (PDF 742 kb). [file 11356_2017_64_MOESM1_ESM.pdf]

How does exposure to pesticides vary in space and time for residents living near to treated orchards?

Hie Ling Wong[1,2], David G. Garthwaite[3], Carmel T. Ramwell[3], Colin D. Brown[1]

[1] Environment Department, University of York, York, YO10 5NG, United Kingdom

[2] Faculty of Earth Science, University Malaysia Kelantan, Locked Bag 100, Jeli, 17600, Kelantan, Malaysia

[3] Fera Science Ltd. (Fera), Sand Hutton, York, YO41 1LZ, United Kingdom

Corresponding author: Hie Ling Wong

e-mail: hw1166@york.ac.uk

telephone number: +44(0) 1904 322999

fax number: +44(0) 1904 322998

Supplementary Material

Table legends

Table S1 Estimated average airborne spray drift (% of sprayed volume) in the bottom 0-2 m layer at different distances that extrapolated based on the measure data at 7.5 m from the last tree row spraying an orchard with a cross-flow fan sprayer and an axial fan sprayer in different growth stages, used in the development of BROWSE model (van de Zande et al. 2014)

Table S2 Default parameter values used in modelling exposure to residents

Table S3 Main stages of apple development and associated interception factors for pesticide applied to the canopy (Jensen and Spliid 2003; Olesen and Jensen 2013)

Table S4 Average monthly temperature between 1980 and 2015 for the regions considered in the study (Met Office 2015)

Table S5 Pesticide active substances with no NO(A)ELs for reproductive and/or developmental effects reported in the literature or reported in concentration unit other than daily exposure

Figure legends

Fig. S1 Map of regions in the UK derived from the Office for National Statistics (2011)

Fig. S2 Sensitivity analysis for the effect of boundary air layers on the emission rates of active substances with low volatility (propyzamide; VP: 5.8×10^{-5} Pa) (a) and medium volatility (chlorpyrifos; VP: 1.43×10^{-3} Pa) (b) from the treated surfaces

Fig. S3 Total amount of pesticide (including tar oils) applied to major orchard crop types between 1987 and 2012 for Eastern (a), West Midlands (b), South-Eastern (c), and South-Western (d) regions. Blanks indicate that none of that orchard type was sampled in that region

F**ig. S4** Usage of pesticide for orchard crop types cultivated in the Eastern, West Midlands, and South-Western regions with usage of tar oils excluded. Data are expressed as number of applications (a) defined as treated area divided by area grown, and average application rate (b) defined as total amount applied divided by number of applications. Here, application is defined as one treatment with one active substance, so successive treatments with a single active substance or a single treatment with a product containing two active substances would both count as two applications

Fig. S5 Aggregated exposures to applied pesticide for residents living 1000 m downwind of individual crop types. Data are shown for four years between 1987 and 2012 for Eastern (a), West Midlands (b), South-Eastern (c), and South-Western (d) regions

Fig. S6 Aggregated hazard quotients based on reproductive/developmental toxicity for pesticide exposure to resident pregnant women living 1000 m downwind of individual crop types. Data are shown for four years between 1987 and 2012 and for Eastern (a), West Midlands (b), South-Eastern (c), and South-Western (d) regions

Fig. S7 Average of total amount of pesticide applied to all crop types in four regions in England and Wales at approximately 4-year intervals between 1987 and 2012

Fig. S8 Total amount of pesticide applied in four regions in England and Wales for 4 years between 1987 and 2012 based on pesticide chemical groups

Fig. S9 Monthly estimates for total amount of pesticide applied to orchards in the South-Eastern region (a) and aggregated exposures for resident pregnant women living 100 m downwind of individual crop types (b) in 1987

Fig. S10 Plot of NO(A)ELs of reproductive and/or developmental toxicity and vapour pressure of individual active substances; plot is divided into approximate quadrants using divisions at 10 mg kg bw^{-1} d^{-1} and $1.0x10^{-5}$ Pa

Fig. S11 Total emission rates of applied pesticides and their respective airborne concentrations at 100 m downwind in four regions in England and Wales for 4 years between 1987 and 2012

Table S1 Estimated average airborne spray drift (% of sprayed volume) in the bottom 0-2 m layer at different distances that extrapolated based on the measure data at 7.5 m from the last tree row spraying an orchard with a cross-flow fan sprayer and an axial fan sprayer in different growth stages, used in the development of BROWSE model (van de Zande et al. 2014)

	Distance from last tree row (m)										
	5	10	15	20	25	30	35	40	45	50	60
Axial fan											
Dormant	74.2	36.9	18.3	9.1	4.5	2.2	1.1	0.6	0.3	0.14	0.03
Intermediate	58.9	28.8	14.1	6.9	3.4	1.6	0.8	0.4	0.2	0.09	0.02
Full leaf	40.4	19.7	9.7	4.7	2.3	1.1	0.6	0.3	0.1	0.06	0.02
Cross-flow fan											
Dormant	76.9	38.0	18.8	9.3	4.6	2.3	1.1	0.6	0.3	0.13	0.03
Intermediate	39.6	19.5	9.6	4.7	2.3	1.1	0.6	0.3	0.14	0.07	0.02
Full leaf	25.6	12.5	6.1	3.0	1.4	0.7	0.3	0.17	0.08	0.04	0.01

Table S2 Default parameter values used in modelling exposure to residents

Parameter	Default value
Adult height, z_r	1.4 m
Body weight, BW	60 kg (for adult as recommended by EFSA, 2014)
Concentration in the turbulent air outside the laminar air layer, C_{air}	Set as zero (= 0 g m^{-3})
Coriolis parameter, f	9.374x10^{-5} s^{-1} (at 40° latitude)
Crop height, h_e	2.0 m (for orchard crop)
Diffusion coefficient in air at 20°C, $D_{a,ref}$	0.43 m^2 d^{-1} (BROWSE[a] definition)
Dry soil bulk density	1.1 g cm^{-3}
Fraction of organic carbon, f_{oc}	0.02 g g^{-1}
$Q10$ factor	1.78 (for every 10°C increase or decrease)
Indirect dermal exposure duration, H	2 hrs (EFSA 2014)
Inhalation rate, IR	13.8 m^3 d^{-1} (for adult as recommended by US EPA)
Inhalation absorption, IA	100% (= 1.0)
Molar enthalpy of evaporation, ΔH_{vap}	95,000 J mol^{-1}
Reference aeric mass of pesticide on the plants, $A_{p,ref}$	1.0 x 10^{-4} kg m^{-2} (= 1 kg ha^{-1})
Soil water content	0.3 g g^{-1}
Transfer coefficient, TC	7,300 cm^2 hr^{-1} (for adult as recommended by EFSA, 2014)
Turf transferable residues, TTR	5 % (= 0.05 for products applied in liquid sprays as recommended by EFSA, 2014)
Treated area	200 x 200 m
Universal gas constant, R	8.314 Pa m^3 K^{-1} mol^{-1}
von Karman's constant, k	0.4
Wind speed, $u(z)$	2.8 m s^{-1} at 2.0 m above the ground (BROWSE[a] definition)

[a] BROWSE refers to the Bystanders, Residents, Operators and WorkerS Exposure models for plant protection products (Butler Ellis et al. 2013)

Table S3 Main stages of apple development and associated interception factors for pesticide applied to the canopy (Jensen and Spliid 2003; Olesen and Jensen 2013)

Apple	Without leaves	Flowering	Leaf development	Full foliage
Month	November-March	April	May-June	July-Oct
CI (%)	50	65	70	80
Fraction on plant	0.5	0.65	0.7	0.8
Fraction on soil	0.5	0.35	0.3	0.2

Table S4 Average monthly temperature between 1980 and 2015 for the regions considered in the study (Met Office 2015)

Month	Eastern	West Midlands	South-Eastern	South-Western
January	4.2	3.9	4.6	4.8
February	4.4	4.0	4.6	4.7
March	6.5	6.1	6.7	6.4
April	8.8	8.2	8.9	8.3
May	11.9	11.2	12.0	11.2
June	14.8	14.1	14.8	13.8
July	17.2	16.3	17.1	15.8
August	17.1	16.0	16.9	15.7
September	14.6	13.6	14.5	13.7
October	11.1	10.2	11.2	10.7
November	7.2	6.7	7.5	7.5
December	4.8	4.4	5.2	5.4

Table S5 Pesticide active substances with no NO(A)ELs for reproductive and/or developmental effects reported in the literature or reported in concentration unit other than daily exposure

No.	Active substance
1	Alloxydim-sodium
2	Benodanil
3	Ditalimfos
4	Nitrothal-isopropyl
5	Nuarimol
6	Propyzamide
7	Pyrifenox
8	Tetradifon

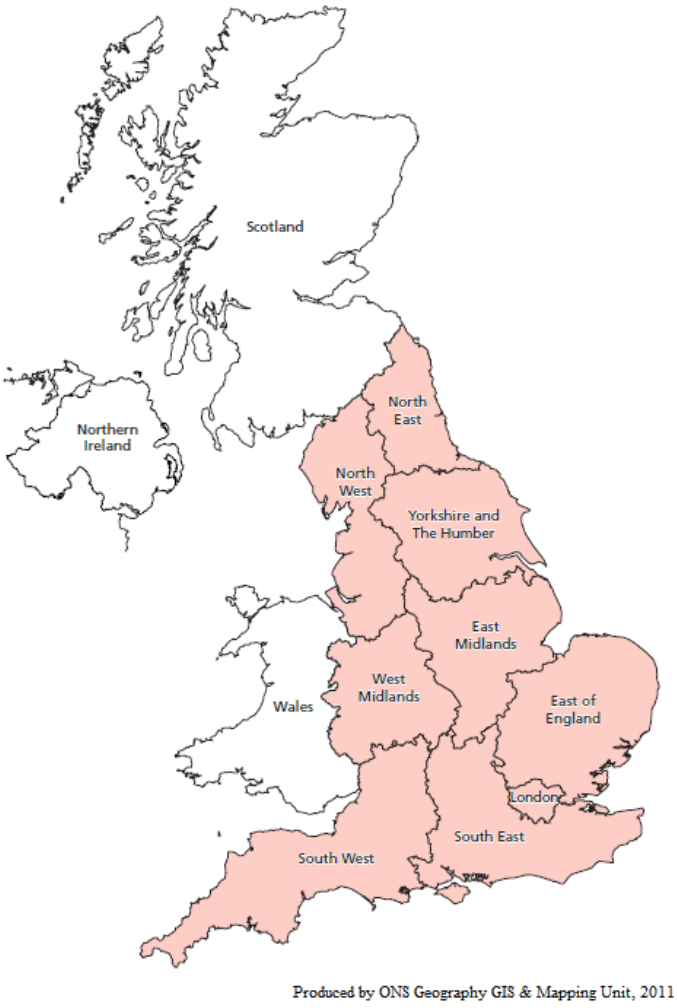

Fig. S1 Map of regions in the UK derived from the Office for National Statistics (2011)

a

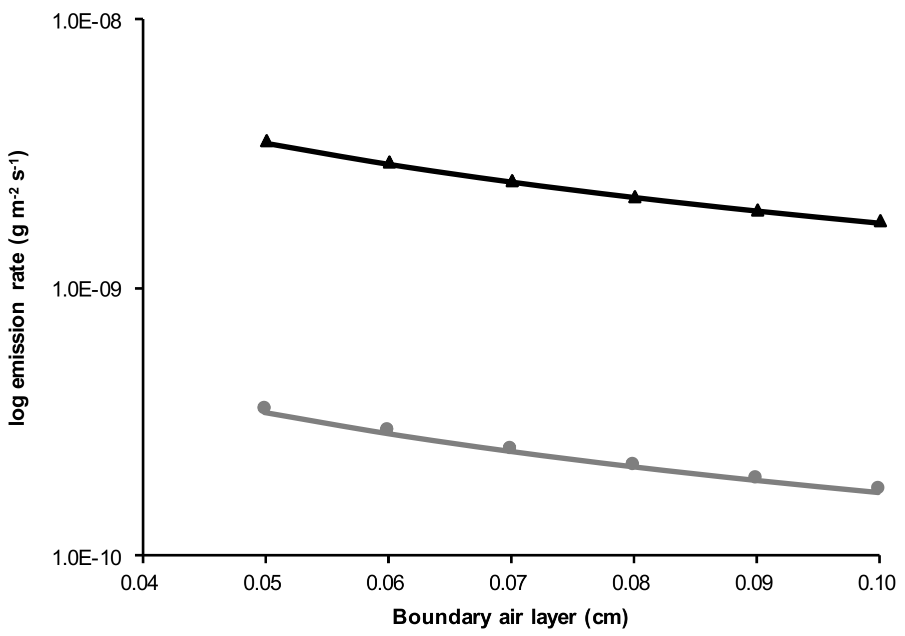

b

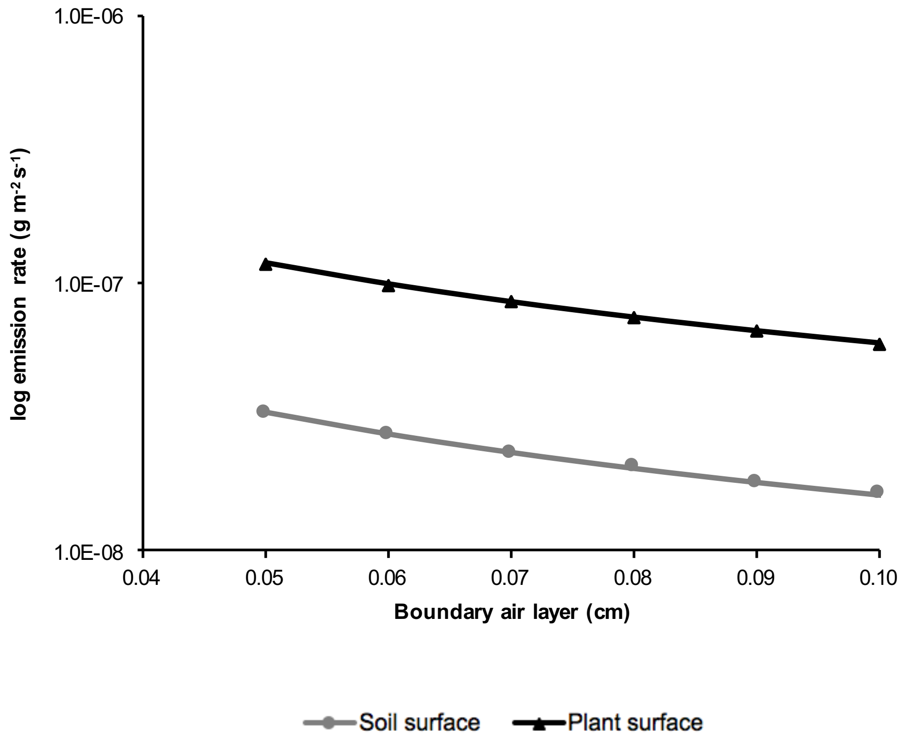

Fig. S2 Sensitivity analysis for the effect of boundary air layers on the emission rates of active substances with low volatility (propyzamide; VP: 5.8×10^{-5} Pa) (a) and medium volatility (chlorpyrifos; VP: 1.43×10^{-3} Pa) (b) from the treated surfaces

a

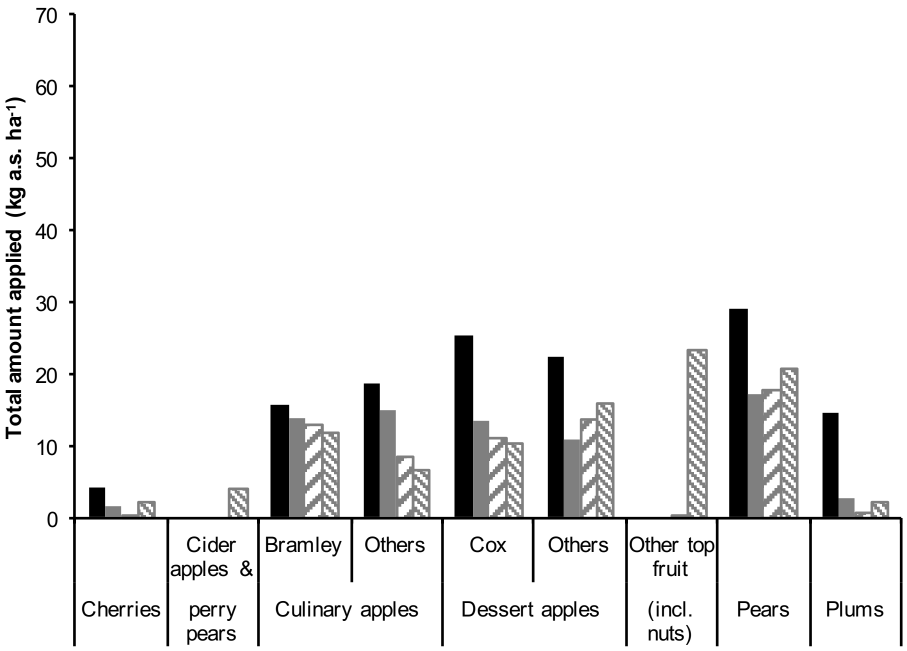
Total amount applied (kg a.s. ha-1)
70
60
50
40
30
20
10
0
Cherries
Cider apples & perry pears
Bramley
Others
Culinary apples
Cox
Others
Dessert apples
Other top fruit (incl. nuts)
Pears
Plums

b

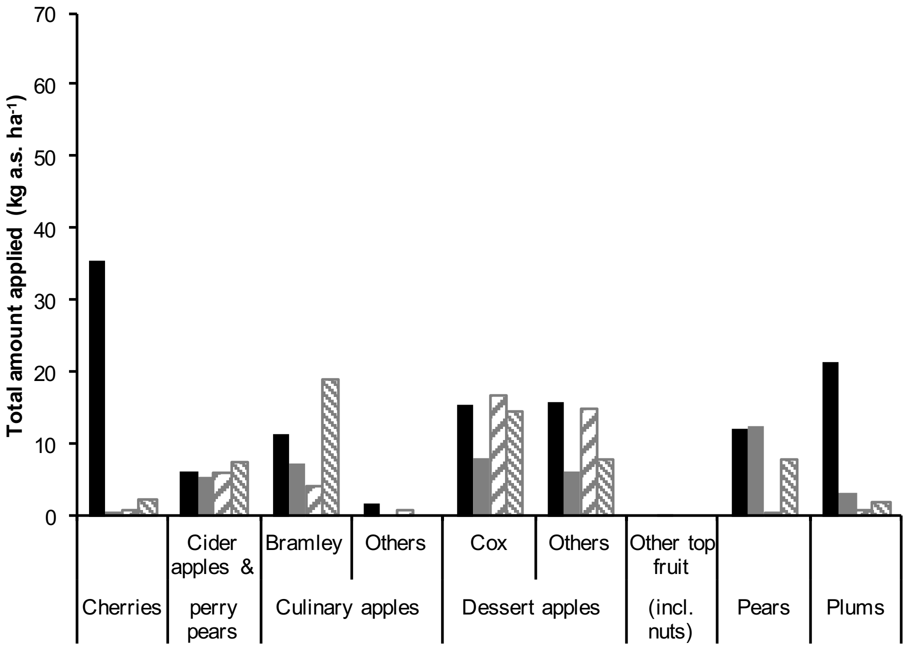
Total amount applied (kg a.s. ha-1)
70
60
50
40
30
20
10
0
Cherries
Cider apples & perry pears
Bramley
Others
Culinary apples
Cox
Others
Dessert apples
Other top fruit (incl. nuts)
Pears
Plums

c

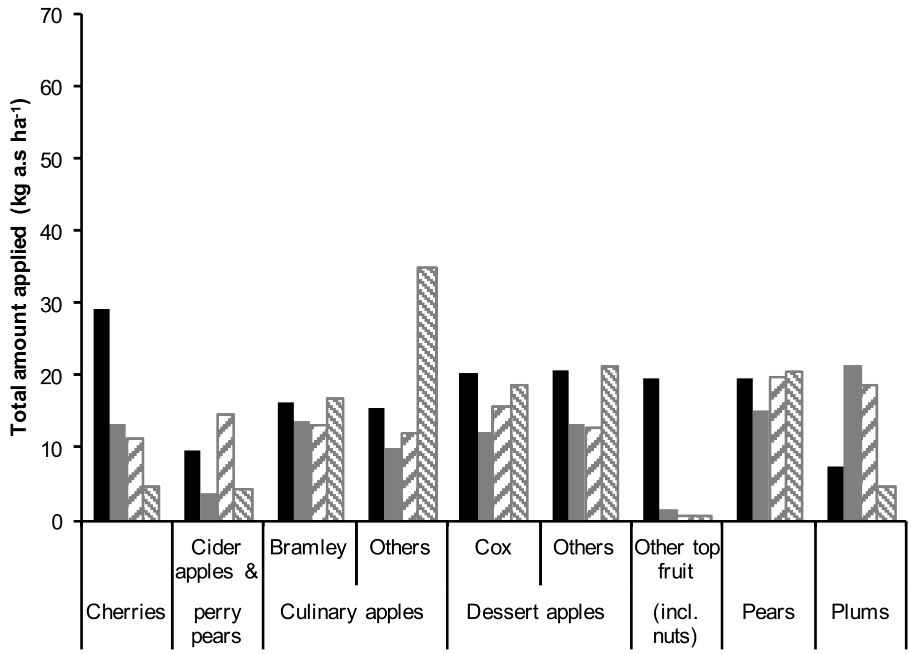

d

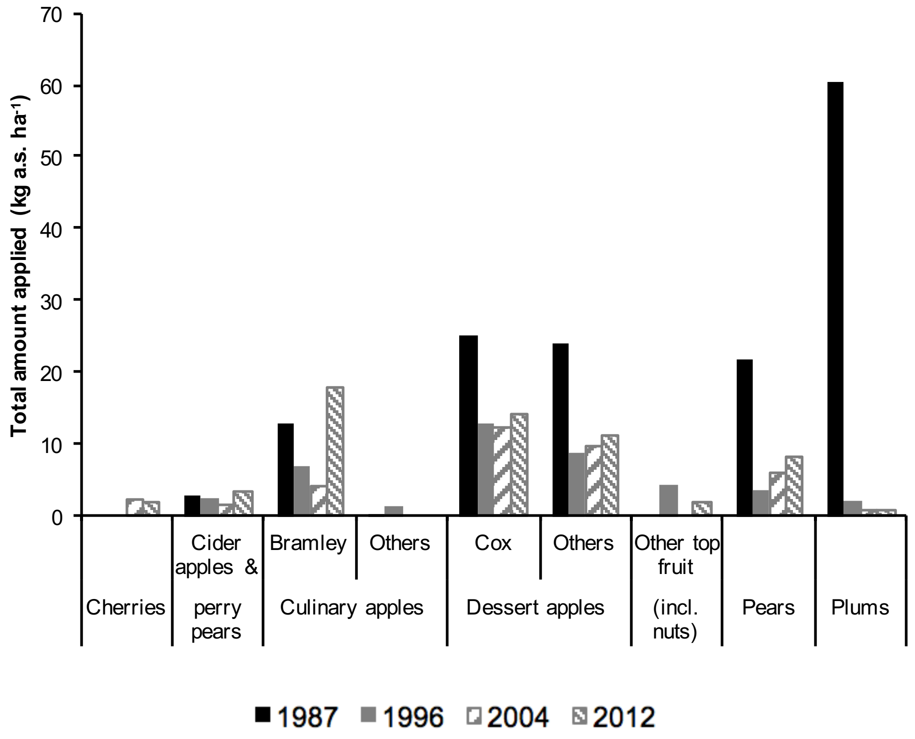

Fig. S3 Total amount of pesticide (including tar oils) applied to major orchard crop types between 1987 and 2012 for Eastern (a), West Midlands (b), South-Eastern (c), and South-Western (d) regions. Blanks indicate that none of that orchard type was sampled in that region

a

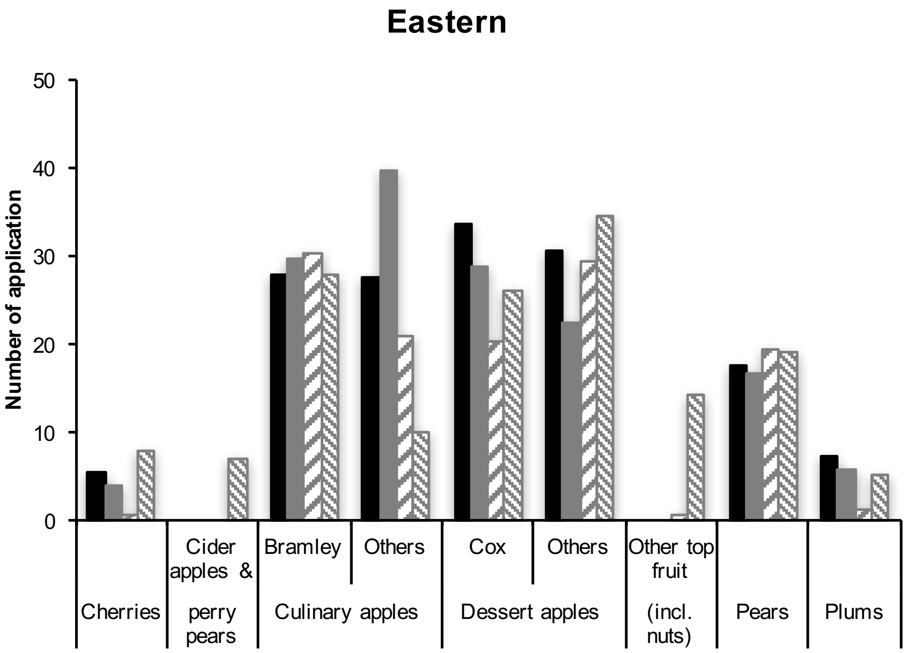

Eastern
Number of application
0
10
20
30
40
50
Cherries
Cider apples & perry pears
Bramley
Others
Culinary apples
Cox
Others
Dessert apples
Other top fruit (incl. nuts)
Pears
Plums

b

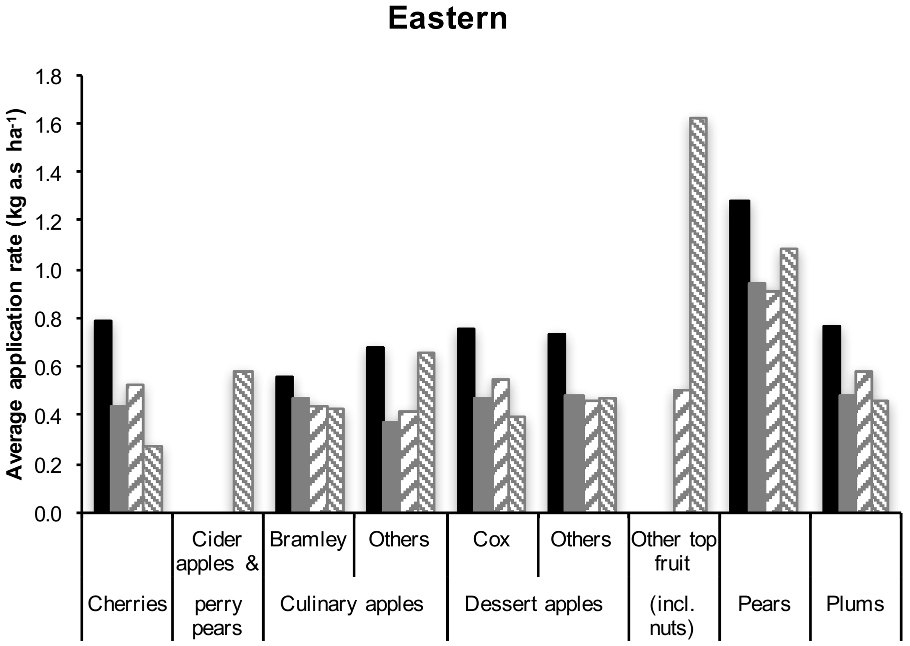

Eastern
Average application rate (kg a.s ha-1)
0.0
0.2
0.4
0.6
0.8
1.0
1.2
1.4
1.6
1.8
Cherries
Cider apples & perry pears
Bramley
Others
Culinary apples
Cox
Others
Dessert apples
Other top fruit (incl. nuts)
Pears
Plums

a

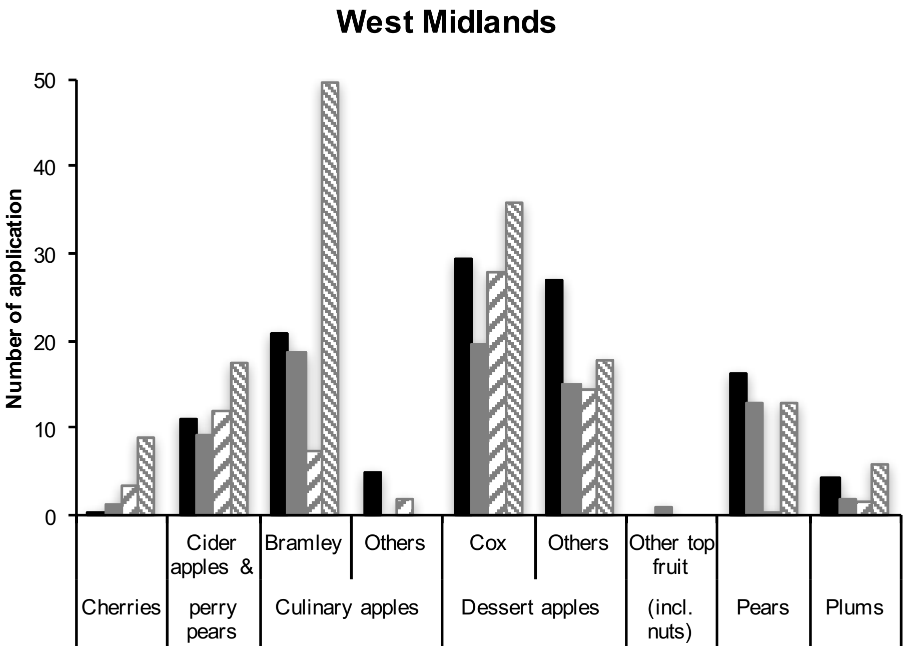

West Midlands
Number of application
0
10
20
30
40
50
Cherries
Cider apples & perry pears
Bramley
Others
Culinary apples
Cox
Others
Dessert apples
Other top fruit (incl. nuts)
Pears
Plums

b

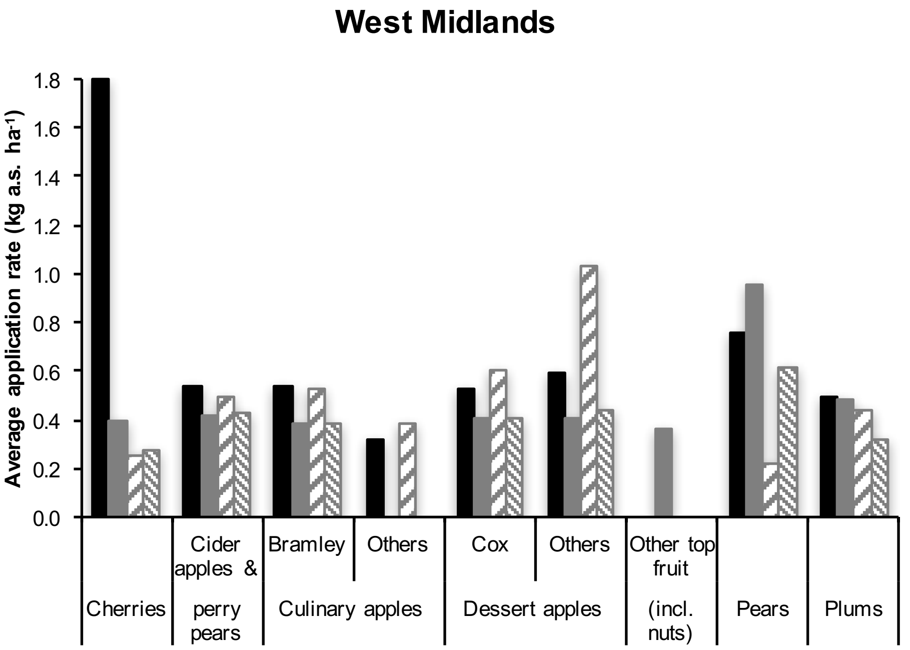

West Midlands
Average application rate (kg a.s. ha-1)
0.0
0.2
0.4
0.6
0.8
1.0
1.2
1.4
1.6
1.8
Cherries
Cider apples & perry pears
Bramley
Others
Culinary apples
Cox
Others
Dessert apples
Other top fruit (incl. nuts)
Pears
Plums

a

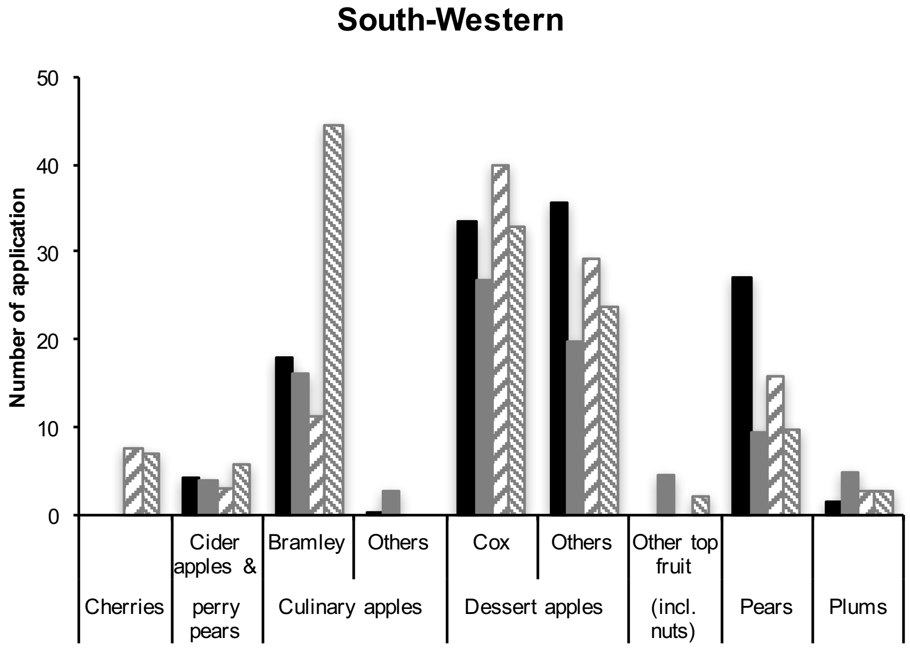

b

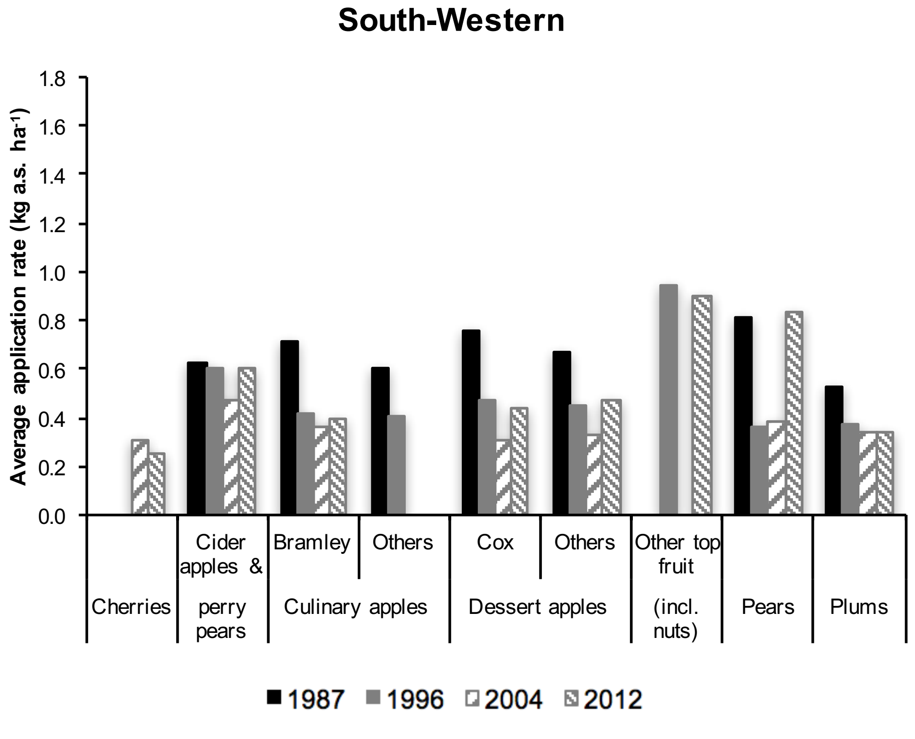

Fig. S4 Usage of pesticide for orchard crop types cultivated in the Eastern, West Midlands, and South-Western regions with usage of tar oils excluded. Data are expressed as number of applications (a) defined as treated area divided by area grown, and average application rate (b) defined as total amount applied divided by number of applications. Here, application is defined as one treatment with one active substance, so successive treatments with a single active substance or a single treatment with a product containing two active substances would both count as two applications

a

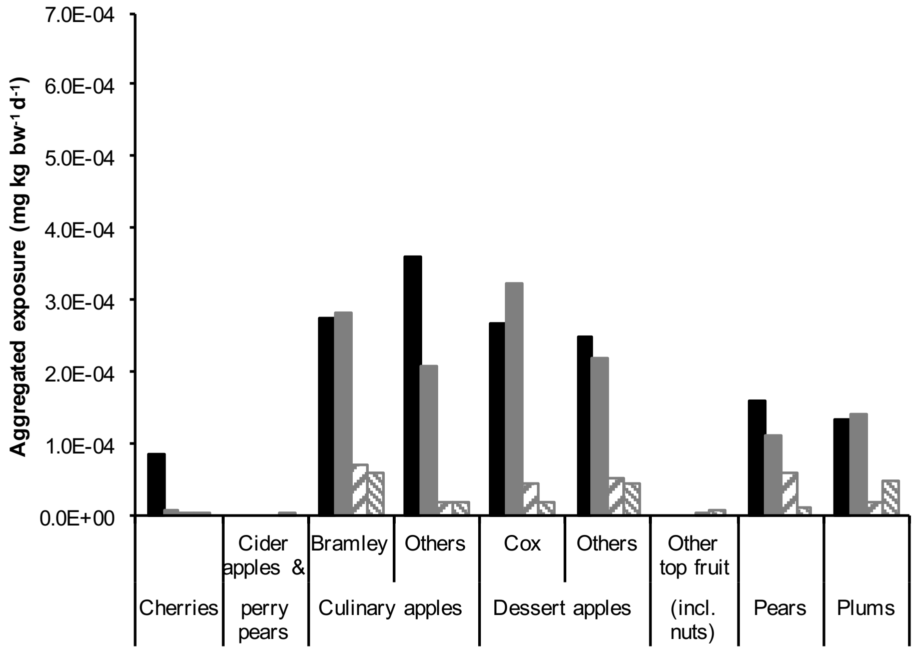

7.0E-04
6.0E-04
5.0E-04
4.0E-04
3.0E-04
2.0E-04
1.0E-04
0.0E+00
Aggregated exposure (mg kg bw-1 d-1)
Cherries
Cider apples & perry pears
Bramley
Others
Culinary apples
Cox
Others
Dessert apples
Other top fruit (incl. nuts)
Pears
Plums

b

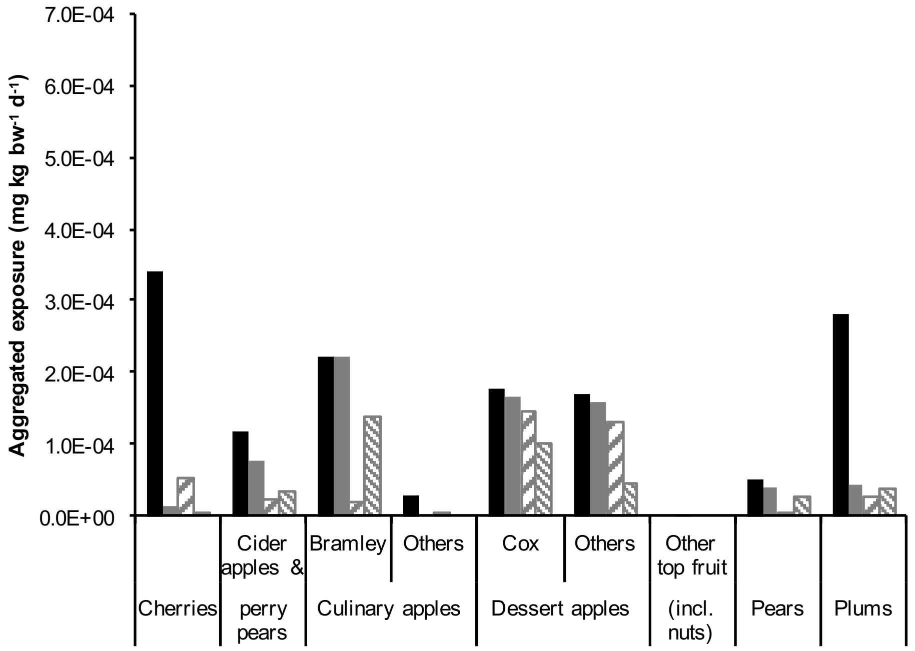

7.0E-04
6.0E-04
5.0E-04
4.0E-04
3.0E-04
2.0E-04
1.0E-04
0.0E+00
Aggregated exposure (mg kg bw-1 d-1)
Cherries
Cider apples & perry pears
Bramley
Others
Culinary apples
Cox
Others
Dessert apples
Other top fruit (incl. nuts)
Pears
Plums

c

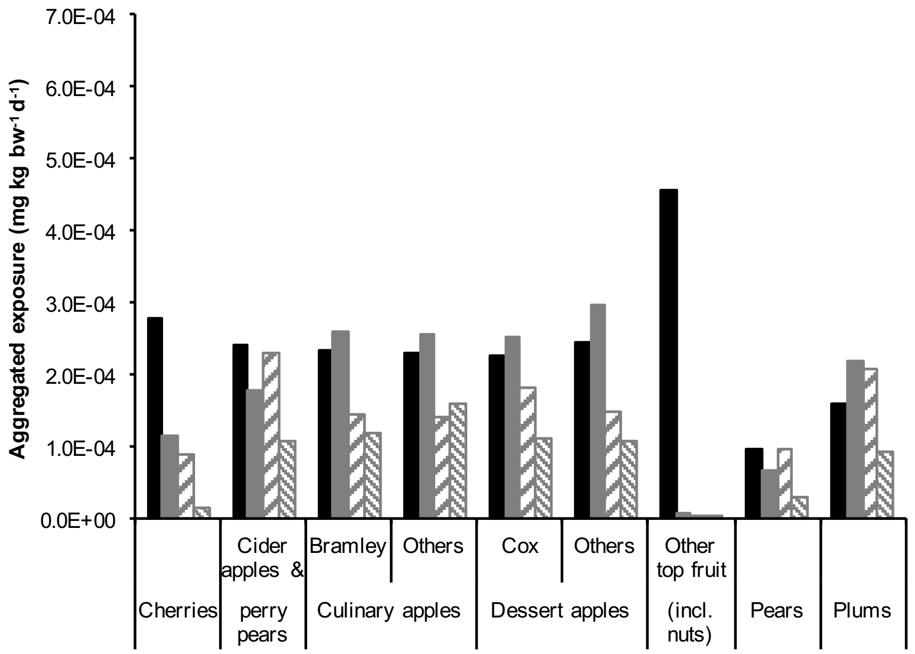

d

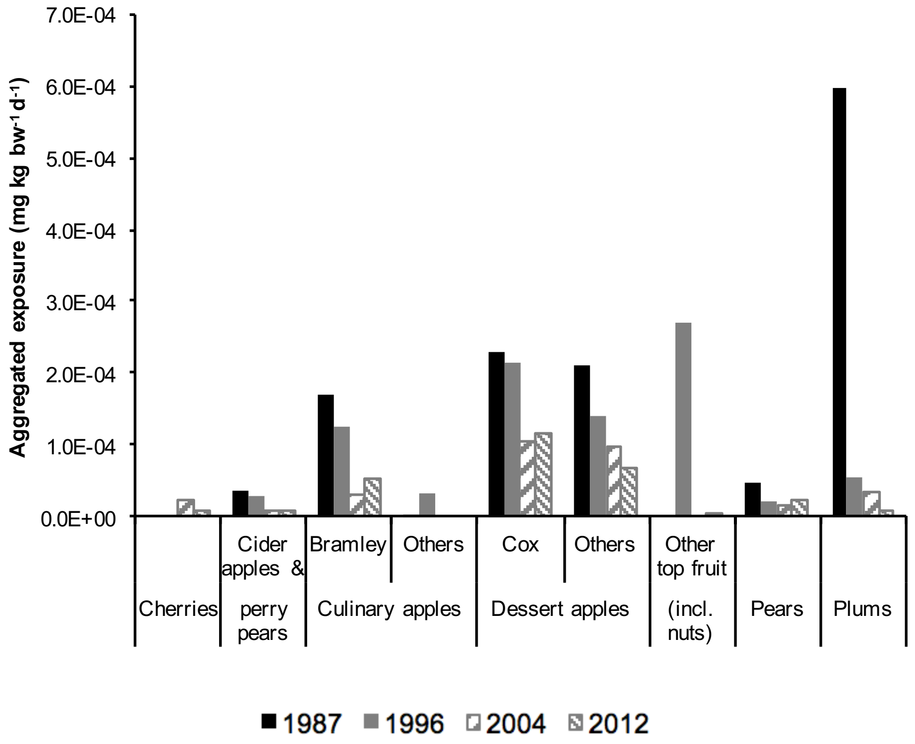

Fig. S5 Aggregated exposures to applied pesticide for residents living 1000 m downwind of individual crop types. Data are shown for four years between 1987 and 2012 for Eastern (a), West Midlands (b), South-Eastern (c), and South-Western (d) regions

a

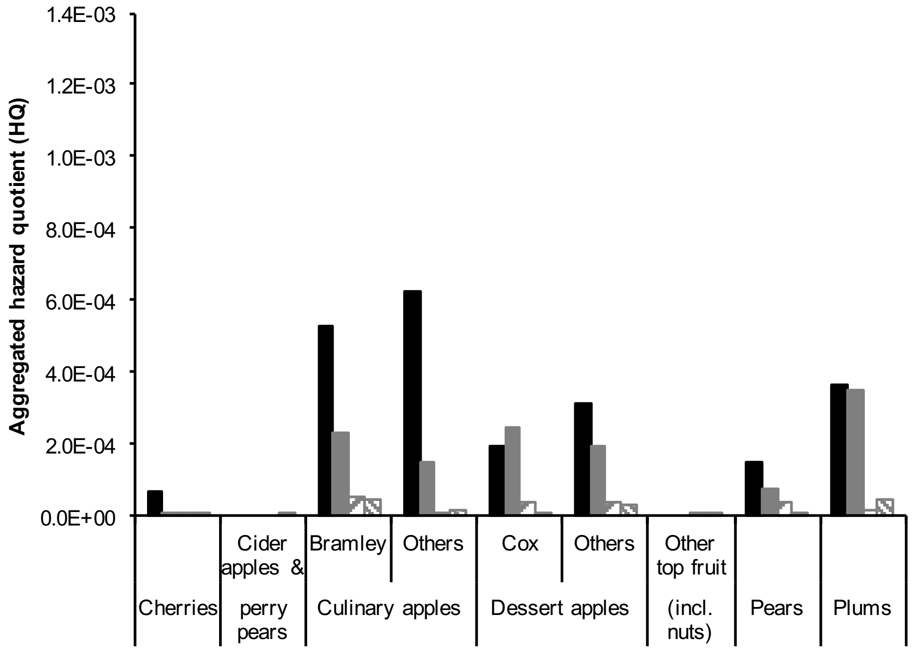
1.4E-03
1.2E-03
1.0E-03
8.0E-04
6.0E-04
4.0E-04
2.0E-04
0.0E+00
Aggregated hazard quotient (HQ)
Cider apples & perry pears
Bramley
Others
Cox
Others
Other top fruit (incl. nuts)
Cherries
Culinary apples
Dessert apples
Pears
Plums

b

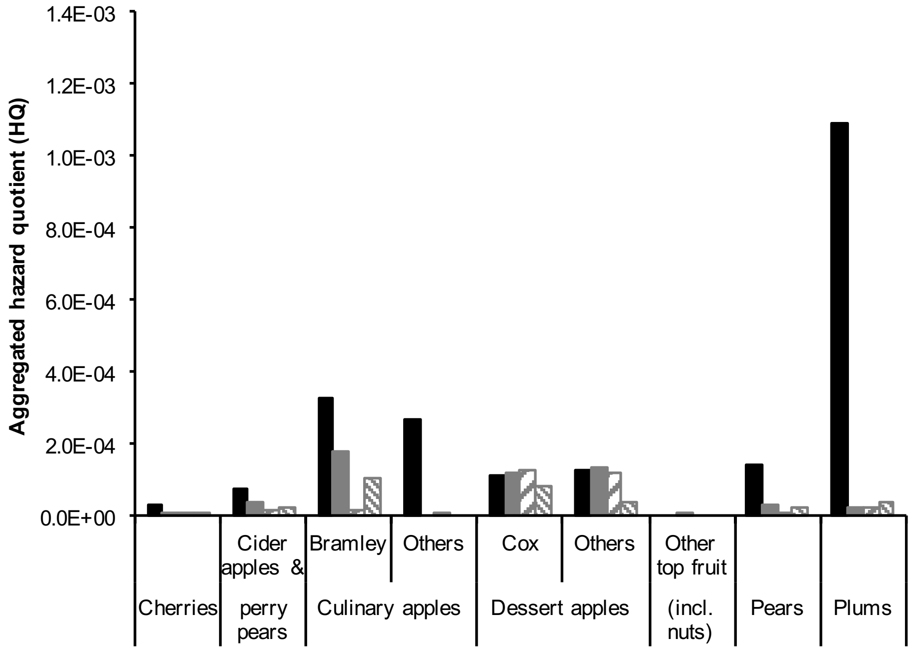
1.4E-03
1.2E-03
1.0E-03
8.0E-04
6.0E-04
4.0E-04
2.0E-04
0.0E+00
Aggregated hazard quotient (HQ)
Cider apples & perry pears
Bramley
Others
Cox
Others
Other top fruit (incl. nuts)
Cherries
Culinary apples
Dessert apples
Pears
Plums

c

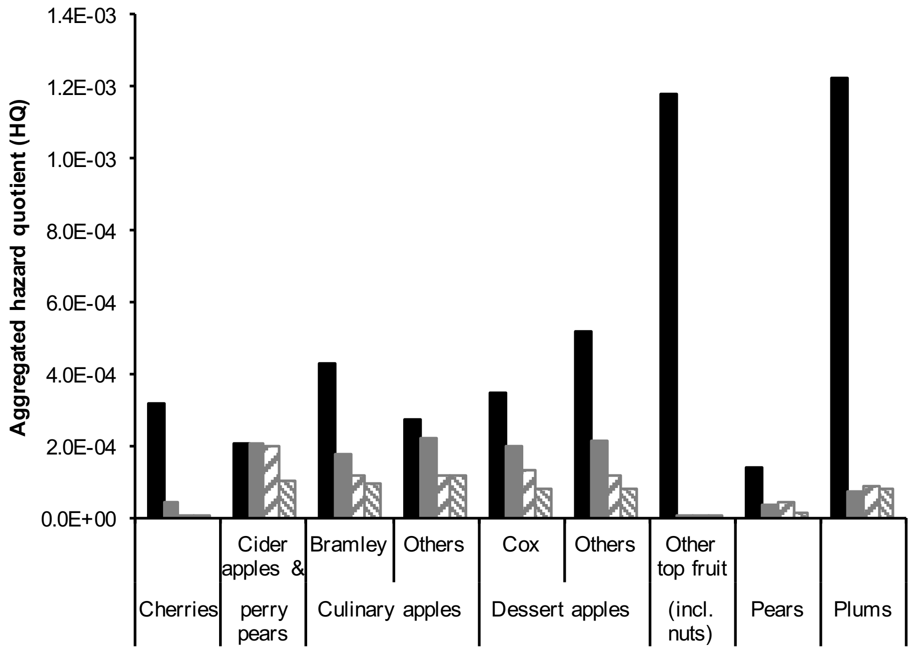

d

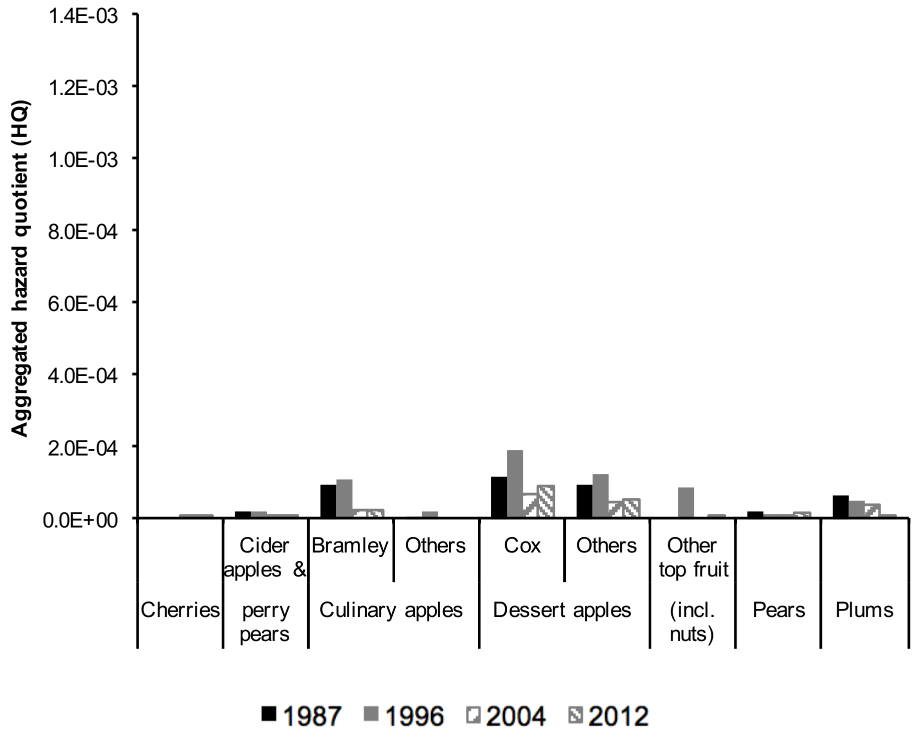

Fig. S6 Aggregated hazard quotients based on reproductive/developmental toxicity for pesticide exposure to resident pregnant women living 1000 m downwind of individual crop types. Data are shown for four years between 1987 and 2012 and for Eastern (a), West Midlands (b), South-Eastern (c), and South-Western (d) regions

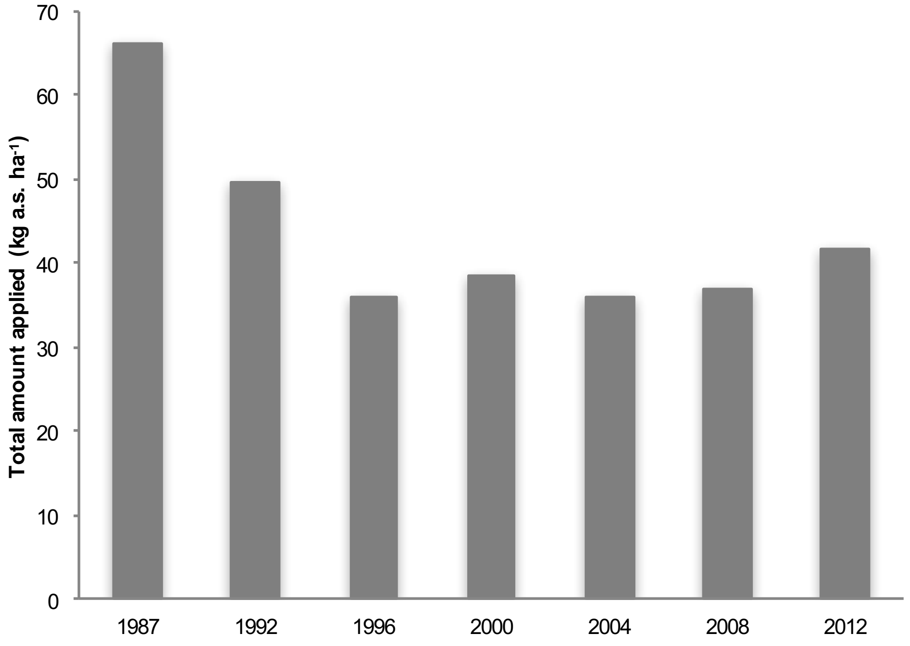

Fig. S7 Average of total amount of pesticide applied to all crop types in four regions in England and Wales at approximately 4-year intervals between 1987 and 2012

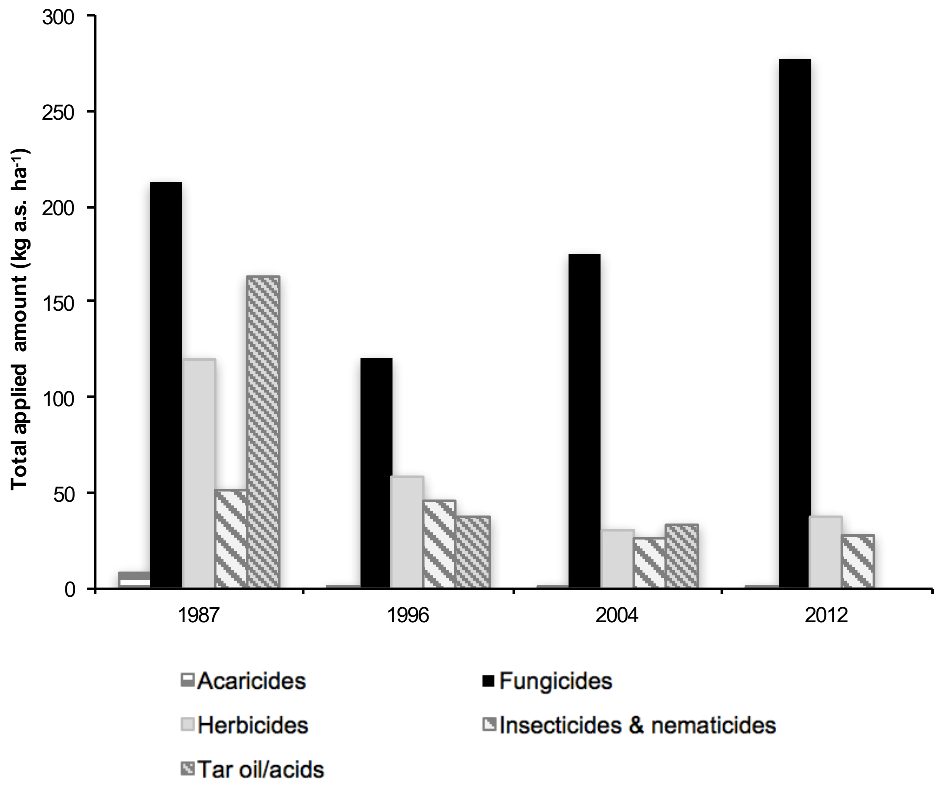

Fig. S8 Total amount of pesticide applied in four regions in England and Wales for 4 years between 1987 and 2012 based on pesticide chemical groups

a

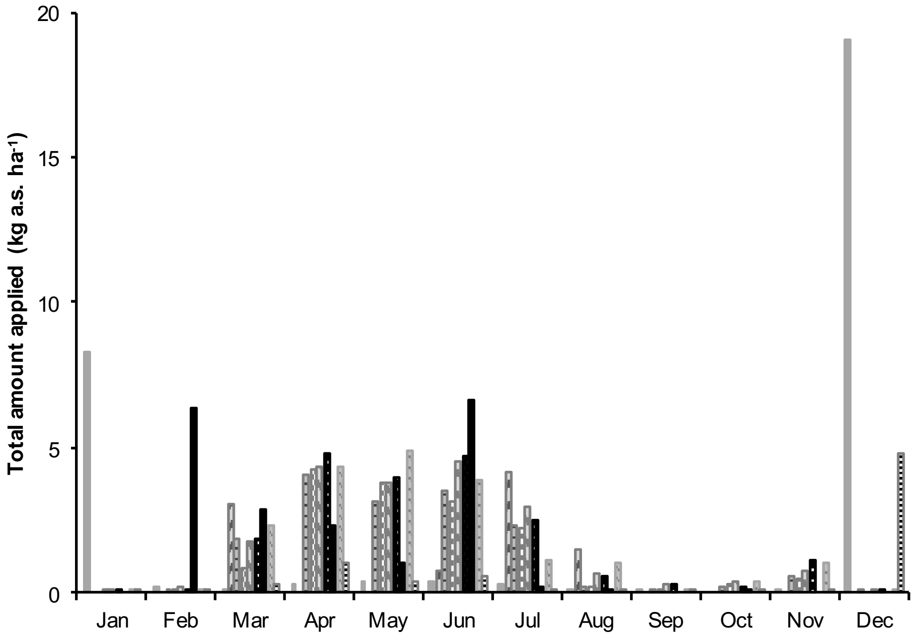

b

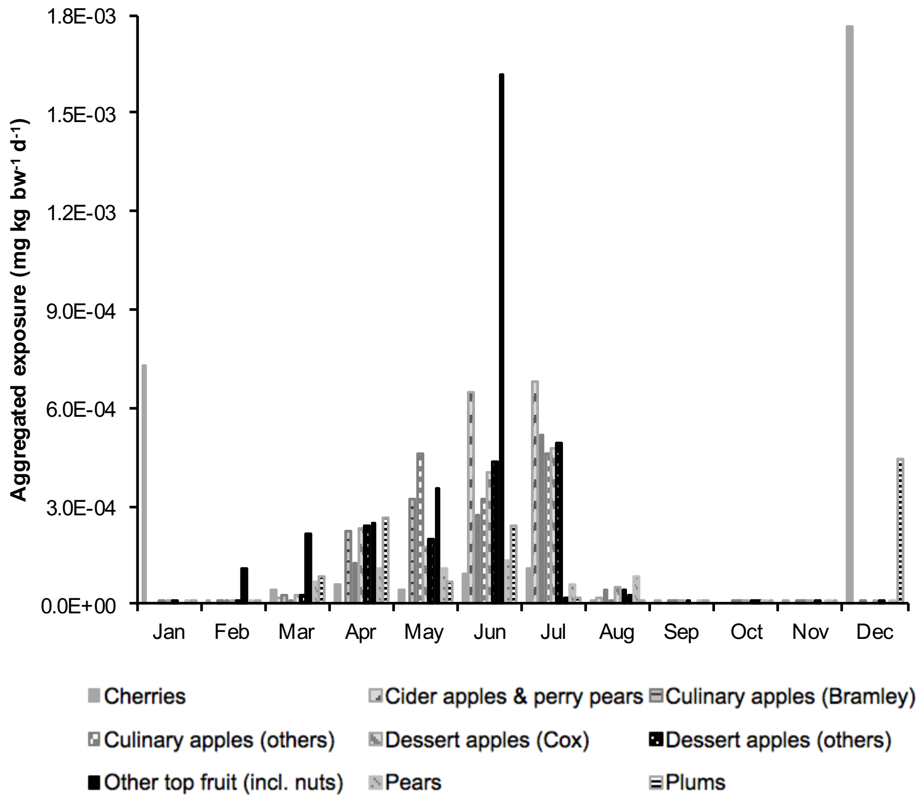

Fig. S9 Monthly estimates for total amount of pesticide applied to orchards in the South-Eastern region (a) and aggregated exposures for resident pregnant women living 100 m downwind of individual crop types (b) in 1987

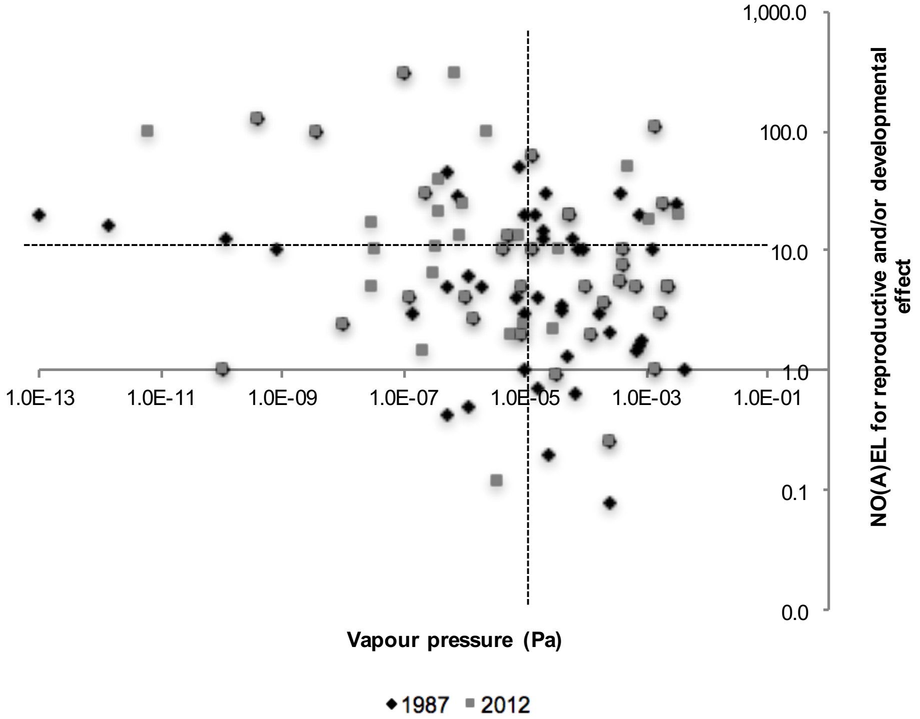

Fig. S10 Plot of NO(A)ELs of reproductive and/or developmental toxicity and vapour pressure of individual active substances; plot is divided into approximate quadrants using divisions at 10 mg kg bw^{-1} d^{-1} and $1.0x10^{-5}$ Pa

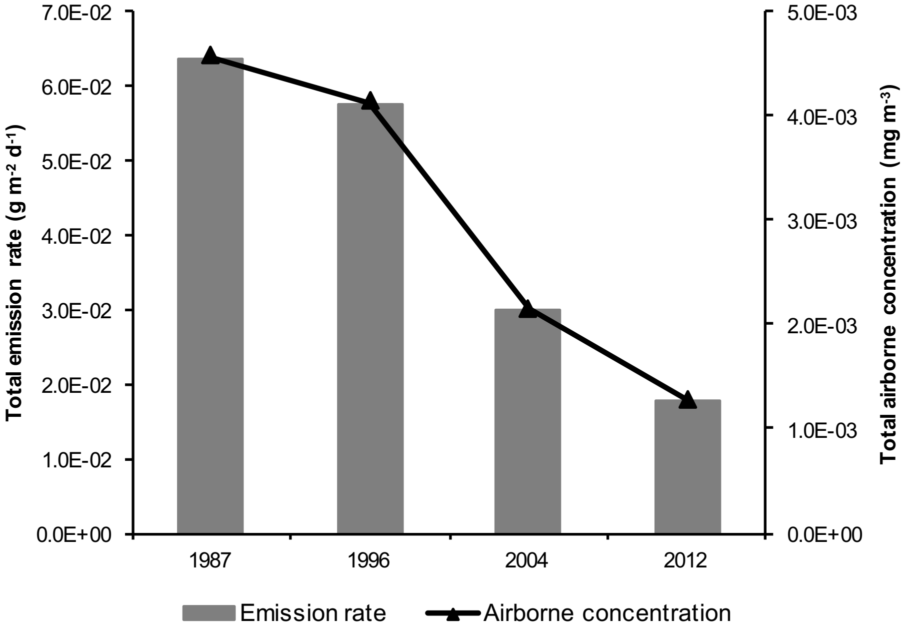

Fig. S11 Total emission rates of applied pesticides and their respective airborne concentrations at 100 m downwind in four regions in England and Wales for 4 years between 1987 and 2012

References

Butler Ellis MC, van den Berg FR, Kennedy MC et al (2013) Work Package 3: Appendix 5 - BROWSE_PEARL-OPS Parameterisation for the BROWSE Exposure Scenarios for Residents and Bystanders. https://secure.fera.defra.gov.uk/browse/software/. Accessed 28 May 2017

Jensen PK, Spliid NH (2003) Deposition of pesticides on the soil surface. Pesticide Research Nr. 65. Danish Environmental Protection Agency, Denmark

Met Office (2015) UK climate: UK and regional series - rank ordered statistics. http://www.metoffice.gov.uk/climate/uk/summaries/datasets. Accessed 1 February 2017

Olesen MH, Jensen PK (2013) External scientific report: collection and evaluation of relevant information on crop interception. EFSA supporting publication 2013:EN-438

Van de Zande JC, Butler Ellis MC, Wenneker M et al (2014) Spray drift and bystander risk from fruit crop spraying. In conference "International advances in pesticide application, Oxford, UK, 8-10 January 2014". Aspects of Applied Biology 122:177-185
